# Antiviral Activity Against Infectious Bronchitis Virus and Bioactive Components of *Hypericum perforatum* L.

**DOI:** 10.3389/fphar.2019.01272

**Published:** 2019-10-29

**Authors:** Huijie Chen, Ishfaq Muhammad, Yue Zhang, Yudong Ren, Ruili Zhang, Xiaodan Huang, Lei Diao, Haixin Liu, Xunliang Li, Xiaoqi Sun, Ghulam Abbas, Guangxing Li

**Affiliations:** ^1^Key Laboratory for Laboratory Animals and Comparative Medicine of Heilongjiang Province, College of Veterinary Medicine, Northeast Agricultural University, Harbin, China; ^2^College of Biological and Pharmaceutical Engineering, Jilin Agricultural Science and Technology University, Jilin, China; ^3^Heilongjiang Key Laboratory for Animal Disease Control and Pharmaceutical Development, Heilongjiang Province, College of Veterinary Medicine, Northeast Agricultural University, Harbin, China

**Keywords:** *Hypericum perforatum* L., infectious bronchitis virus, antiviral activity, melanoma differentiation-associated protein 5, nuclear factor kappa beta, high-performance liquid chromatography/electrospray ionization-mass spectroscopy

## Abstract

*Hypericum perforatum* L., also known as *Saint John’s Wort*, has been well studied for its chemical composition and pharmacological activity. In this study, the antiviral activities of *H. perforatum* on infectious bronchitis virus (IBV) were evaluated *in vitro* and *in vivo* for the first time. The results of *in vitro* experiments confirmed that the antiviral component of *H. perforatum* was ethyl acetate extraction section (HPE), and results showed that treatment with HPE significantly reduced the relative messenger ribonucleic acid (mRNA) expression and virus titer of IBV, and reduced positive green immunofluorescence signal of IBV in chicken embryo kidney (CEK) cells. HPE treatment at doses of 480–120 mg/kg for 5 days, reduced IBV induced injury in the trachea and kidney, moreover, reduced the mRNA expression level of IBV in the trachea and kidney *in vivo*. The mRNA expression levels of IL-6, tumor necrosis factor alpha (TNF-α), and nuclear factor kappa beta (NF-κB) significantly decreased, but melanoma differentiation-associated protein 5 (MDA5), mitochondrial antiviral signaling gene, interferon alpha (IFN-α), and interferon beta (IFN-β) mRNA levels significantly increased *in vitro* and *in vivo*. Our findings demonstrated that HPE had significant anti-IBV effects *in vitro* and *in vivo*, respectively. In addition, it is possible owing to up-regulate mRNA expression of type I interferon through the MDA5 signaling pathway and down-regulate mRNA expression of IL-6 and TNF-α *via* the NF-κB signaling pathway. Moreover, the mainly active compositions of HPE analyzed by high-performance liquid chromatography/electrospray ionization–mass spectroscopy (ESI-MS) are hyperoside, quercitrin, quercetin, pseudohypericin, and hypericin, and a combination of these compounds could mediate the antiviral activities. This might accelerate our understanding of the antiviral effect of *H. perforatum* and provide new insights into the development of effective therapeutic strategies.

## Introduction

The infectious bronchitis virus (IBV) is a prototype coronavirus containing a single-stranded positive-sense RNA genome (Cook et al., 2012). IBV is the etiologic agent of infectious bronchitis (IB), which is a highly contagious, acute viral respiratory disease of chickens. IBV has been reported by many researchers all over the world (Jungherr and Terrells, 1948; Jungherr et al., 1956; Fabricant, 1998; Yu et al., 2001; Benyeda et al., 2009; Sjaak De Wit et al., 2011; Westerbeck and Machamer, 2019; Wu et al., 2019). IBV has led to severe losses in the poultry industry (Jordan, 2017), the direct losses are due to highly mortality, poor egg quality, and meat production, and the indirect losses result in increased costs and challenges in IBV prevention (Liang et al., 2019). At present, live attenuated vaccines are widely used for the prevention and control of IB. However, due to extensive genetic diversity of IBV strains, the vaccines are becoming increasingly inefficient, with poor cross-protection effects among different serotypes of vaccines (Mo et al., 2013; Chen et al., 2015; Lin and Chen, 2017; Yan et al., 2018). Meanwhile, due to the lack of coordinated effort to prevent the IBV, and the lack of proper surveillance plus the introduction of foreign strains to combat the IBV in certain regions, the prevention and control of IBV has become very difficult. Therefore, it is imperative to find an effective antiviral drug or agent for the prevention of IBV.

In order to control drug residues, the Chinese government has banned the use of antiviral drugs in food animals in China. Therefore, the use of traditional antiviral herbs with no obvious side effects on the human body is still a major focus. Some reports have confirmed that traditional Chinese herbs could effectively inhibit the infection and replication of various viruses (Li et al., 2009; Wang et al., 2015; Choi et al., 2016; Sun et al., 2016; Choi et al., 2017; Yin et al., 2017; Yi et al., 2018; Luo et al., 2019). *Hypericum perforatum* L. belongs to the genus *Guttiferae*, which contains approximately 400 species all over the world. The extract of *H. perforatum* contains several active compounds, including flavonoids, naphthodianthrones, and phloroglucinol derivatives (Napoli et al., 2018; Barnes et al., 2019). Several reports have shown that *H. perforatum* extract had antiviral effects, such as influenza A virus, porcine respiratory and reproductive syndrome virus (PRRSV), and HIV (Barnes et al., 2001; Birt et al., 2009; Pu et al., 2009a; Pu et al., 2009b; Pu et al., 2012). Like influenza A virus and PRRSV, IBV also belongs to RNA virus, but these PRRSV and IBV belong to different viral families. Since *H. perforatum* could resist influenza A virus and PRRSV, could it resist IBV?

In this study, we investigated the antiviral effects of *H. perforatum* extract against IBV utilizing several approaches *in vitro* and *in vivo* for the first time. Moreover, the purpose of this work was to point out the antiviral active ingredients of *H. perforatum* and its anti-IBV mechanisms. For this purpose, the relative messenger ribonucleic acid (mRNA) expression levels of IBV in CEK cells, tracheas, and kidneys were measured. The positive green immunofluorescence signal of IBV in CEKs was observed. In addition, hematoxylin-eosin (HE) staining of tracheas and kidneys was performed, and the relative mRNA expression of IL-6, tumor necrosis factor alpha (TNF-α), IFN-α, IFN-β, MDA5, MAVS, and nuclear factor kappa beta (NF-κB) *in vitro* and *in vivo* were analyzed. Finally, the antiviral principal chemical composition of *H. perforatum* extract was analyzed by high-performance liquid chromatography (HPLC)/ESI-MS. In summary, our findings for the first time showed that *H. perforatum* extract had significant antiviral effect on IBV, and it might up-regulate mRNA expression levels of type I interferon *via* MDA5 pathway and down-regulate mRNA expression levels of IL-6 and TNF-α through the NF-κB pathway. The HPE was found to be composed mainly of hyperoside, quercitrin, quercetin, pseudohypericin, and hypericin, and a combination of these compounds could mediate the antiviral activities.

## Materials and Methods

### Virus and Chicken Embryos

The IBV M41 strain (GenBank: FJ904723.1) was provided by Key Laboratory for Laboratory Animals and Comparative Medicine, Northeast Agricultural University and propagated in 10-day-old specific pathogen-free (SPF) chicken embryos (Harbin Veterinary Research Institute, CAAS). 100 µl IBV M41 strain was inoculated into SPF chicken embryos under aseptic operation. The survival status of chicken embryos was observed every 12 h, and the chicken embryos that died within 24 h were abandoned. Then allantoic fluid from infected embryos was collected at 72 h post-inoculation and stored at −80°C.

### Reagents and Animals

*H. perforatum* was picked from Tongjiang County, Sichuan Province (east longitude 106°67′–107°73,′ north latitude 32°18′–33°71,′ China) and authenticated by Professor Zhongbao Zhang (Jilin Agriculture Science and Technology College, Jilin, PR China). The reference specimen (voucher number, 6184) (**Supplementary Figure 1**) was deposited at Herbarium (herbarium code: PE), Institute of Botany, Chinese Academy of Sciences (no. 20, Xiangshan Nanxincun, Haidian District, Beijing, China). Acetonitrile for HPLC was purchased from Merck (Darmstadt, Germany). Other solvents used for extract preparation, such as methanol and ethyl acetate were analytical grade and obtained from Tianjin Reagent Company (Tianjin, PR China). Ribavirin (RT, H51023508) was purchased from Sichuan Baili Pharmaceutical Co., Ltd., Chengdu, China. The standard compounds of hyperoside, quercitrin, quercetin, pseudohypericin, and hypericin were purchased from National Institute for the Control of Pharmaceutical and Biological Products in China. Because hypericin, the main active component of *H. perforatum*, is sensitive to light, all the experiments in this study were carried out under the condition of avoiding light or low light.

One hundred and forty four 15-day-old (SPF) chickens were purchased from Harbin Veterinary Research Institute, Heilongjiang Province, China. Experimental procedures were approved by the Institutional Animal Ethical Committee of Northeast Agricultural University (no. SRM-08). All animal studies were complied with the animal experiment guidelines of the Animal Experimentation Ethics Committee of Northeast Agricultural University.

### Preparation of the Crude Extract

The crude extract of *H. perforatum* was prepared in the laboratory. In brief, the drug was pretreated with carbon dioxide supercritical extraction method, and the supercritical extraction extract (SEE) was obtained with a yield of 8.1% (wt/wt). Then, 15 g of the pretreated medicinal material was weighed, 1.0 L of 85% methanol was added (Raclariu et al., 2017), ultrasonic extraction was performed twice for 30 min, and ultrasonic power was 400 W. The two extract solutions were combined and filtered, and concentrated under reduced pressure to obtain the crude extract with a yield of 22.4% (wt/wt).

### Fractionation of the Crude Extract

Approximately 6 g of the crude extract of *H. perforatum* was dissolved in 200 ml of distilled water. An equal volume of ethyl acetate was added before shaking vigorously and separating in a separating funnel. The ethyl acetate layer (upper layer) and the water layer (lower layer) were obtained. It was then extracted with ethyl acetate until the upper layer had no color. All ethyl acetate extractions and water extractions were combined and evaporated to obtain extract of *H. perforatum* ethyl acetate (HPE) and extract of *H. perforatum* water (HPW).

A stock solution of HPE (200 mg/ml), HPW (200 mg/ml) and SEE (200 mg/ml) was prepared with dimethylsulfoxide (DMSO, Sigma), respectively. Immediately before each experiment *in vitro*, the working solution of *H. perforatum* extract was diluted in M199 medium without serum to obtain a final concentration. The HPE was dissolved in 5% DMSO, while ribavirin (RT) was dissolved in physiological saline solution (PSS, 0.86% NaCl) *in vivo* experiments.

### Adaptation and Replication of Infectious Bronchitis Virus in Chicken Embryo Kidney Cells

CEK cells were primary cultured from 18-day-old SPF chicken embryo (Harbin Veterinary Research Institute, CAAS) and prepared according to the standard technique (Schat and Purchase, 1998; Ghetas et al., 2015). The cells were cultured in M199 medium (Thermo Fisher Scientific, USA) supplemented with penicillin and streptomycin, and 10% serum at 37°C with 5% CO_2_. Then, IBV M41 strain was passaged in CEK cells for adaption. 500 µl IBV M41 allantoic fluid was inoculated into CEK cells in 5 ml cell culture flask, and the cytopathic effect (CPE) was observed. When the cells showed obvious typical CPE, the time and degree of the lesions were recorded and the first generation of IBV was collected by freezing and thawing cells repeatedly. Then, the collected first generation IBV was inoculated into CEK cells according to the above method, and the time and lesion degree of CPE were also recorded. The second generation IBV was collected according to the above method. After 10 times of passages of IBV in CEK cells, there appeared typical CPE of cell exfoliation and fusion at same time post inoculation regularly. IBV was collected and stored at −80°C until use. The results of adaptation and replication in CEK cells, such as CPE, reverse transcription polymerase chain reaction (RT-PCR), and IBV growth curve determined by tissue culture infective dose (TCID_50_) at different time were tested.

### Cytotoxicity Assay

The cytotoxicity test was carried out according to the published method of thiazolyl blue tetrazolium bromide (MTT), and minor modifications were made (Sui et al., 2010). The monolayer of CEK cells plated on 96-well culture plate were washed with D-Hanks solution three times, and then HPE, HPW, and SEE with concentrations of 39.06, 78.13, 156.25, 312.50, and 625.00 µg/ml were added to the hole (three repeated holes per concentration). The control cells were incubated in the absence of experimental compounds, but incubated with the same concentration of DMSO. The CEK cells were cultured at 37°C for 48 h, then 20 μl MTT with concentrations of 5 mg/ml in M199 medium were added, and then the cells were incubated at 37°C for 4 h. After washing the CEK cells with D-Hanks, 200 μl DMSO was added to each well, then the CEK cells were incubated at 37°C for 10 min, and shaken gently at room temperature for 10 min to dissolved formazan precipitates, and OD_570_ was determined. Using the mean values, the cell survival rate is calculated according to the following formula:

(1) $$\text{survival rate}=\frac{OD_{570}\mathrm{drug}}{OD_{570}\mathrm{control}}\times \text{100\% }$$

### Antiviral Activity Assays

#### Pre-Treatment of Cells Prior to Infection

In order to analyze the effects of HPE, HPW, and SEE on cells, CEK cells cultured on 96 well plates were incubated with HPE, HPW, and SEE solutions at concentrations of 78.13, 39.06, 19.53 μg/ml at 37°C for 2 h, respectively, and then washed with D-Hanks for three times. The CEK cells were then infected with 100 TCID_50_ IBV and cultured at 37°C for 30 h. The cell samples were frozen and thawed repeatedly for three times, and the total RNA was extracted and reverse transcribed into complementary deoxyribonucleic acid (cDNA). The relative mRNA expression of IBV N gene was detected by real-time quantitative RT-PCR (qRT-PCR). In addition, according to the conventional method, the virus titer of cell samples was determined by TCID_50_. The specific operations were as follows: CEK cells were cultured in 96-well tissue culture plates, and when the cells were full of monolayer, IBV virus was diluted to 10^−1^, 10^−2^, 10^−3^…, and 10^−12^ times with serum-free M199, respectively. Then the monolayer CEK cells were washed with D-Hanks solution three times, and the D-Hanks solution was discarded. Then the 100 µl IBV virus diluent as mentioned above was added to each hole, and each virus diluent repeated eight holes. The cells were treated by the same method with M199 culture as normal control. The culture plate was cultured in 5% CO_2_ and 37°C for 48 h. The cytopathic effect (CPE) was observed every 24 h, and the number of CPE pores and no CPE pores were recorded. According to the Reed-Muench method as previously described (Guo et al., 2016; Yang et al., 2018b), the TCID_50_ was calculated according to the number of CPE holes recorded. At the same time, the infected CEK cells treated with 10 μg/ml RT and CEK cells infected with IBV as control.

### Direct Treatment of Virus-Infected Cells

In order to determine the impact of HPE, HPW, and SEE on IBV-infected cells, the CEK cells cultured on 96 well plates were infected with 100 TCID_50_ IBV at 37°C for 2 h, and then treated with HPE, HPW, and SEE solution at concentrations of 78.13, 39.06, 19.53 μg/ml at 37°C for 30 h. According to the above description, the relative mRNA expression level and virus titer of IBV were detected.

### Pre-Treatment of Virus Prior to Infection

In order to study the direct effects of HPE, HPW, and SEE on the virus, 100 TCID_50_ IBV was incubated with 78.13, 39.06, 19.53 μg/ml HPE, HPW, and SEE solution at 37°C for 2 h, respectively. Then CEK cells were infected with the drug-treated IBV at 37°C for 30 h. As described above, the relative mRNA expression levels and the virus titer of IBV were detected.

### Immunofluorescence Assay

In the above three experimental designs, the best antiviral way was selected for indirect immunofluorescence assay. After washing CEK cells with PBS, fixed with 4% paraformaldehyde, then incubated with 0.1% glycine and quenched with 1% Triton-X 100 for 10 min. After washing CEK cells with PBS for 3 times, the CEK cells were incubated with rabbit anti-IBV antibody (1:200) (prepared and stored in Laboratory of Pathology and Anatomy, Northeast Agricultural University) for 1 h, and then incubated with fluorescent labeled goat anti-rabbit IgG (1:500) (Zhongshan, China) for 30 min in the dark. The fluorescent images were examined with a fluorescence microscope (Ti-S, Nikon, Japan). At the same time, the infected cells treated with 10 μg/ml RT, and CEK cells infected with IBV, and the mock CEK cells were set as controls.

### Infection Model and Drug Treatment

One hundred and forty four 2-week-old SPF chicks were randomly divided into HPE treated high dose group (HPE-TH), HPE treated middle dose group (HPE-TM), HPE treated low dose group (HPE-TL), the IBV infected group (IBV), the ribavirin treated (RT) group, and normal control (NC) group with 24 chicks in each group. Each group was completely isolated from the other groups, and housed in negative pressure-filtered air isolators under pathogen-free conditions. Chicks from in three HPE-treated groups, the IBV-infected group, and the RT group were inoculated by nasal drops with 0.1 ml of 100 EID_50_ of IBV M41 allantoic fluid per chick. Chicks in the control group were inoculated with 0.1 ml sterilized negative allantoic fluid in the same manner. The chicks of the three HPE groups and the RT group were orally administered varying doses of three HPE (480 mg/kg, 240 mg/kg, 120 mg/kg) and ribavirin (60 mg/kg), respectively. Both agents were administered twice daily for 5 days, beginning at 24 h after exposure to the virus. At the same time, the chicks of the IBV-infected group and the normal group were orally administered with PSS. At 2, 4, 6, and 8 days post-infection, six chicks in each group were bled before euthanasia and necropsy at each designed day. The trachea and kidney tissues were collected and immediately extracted to obtain the total RNAs. At the same time, tracheal and kidney in each group of chickens at 6 days postinfection were fixed in 10% buffer formaldehyde for histopathological assay.

### Histopathology Assay

After washing in phosphate-buffered saline (PBS), the trachea and kidney tissues were fixed in 10% formaldehyde solution for 7 days, embedded in paraffin wax, cut into 3-μm-thick sections (Leica RM4450, Germany), and stained with HE using standard histological staining procedure. The slides were examined by light microscopy (Nikon, Japan). The tissue slides were examined and evaluated according to the previous histopathologic scoring method for trachea (Levisohn et al., 1986; Todd et al., 1991) and kidney (Reinhard et al., 1991; Laudert et al., 1993; Canales et al., 2012) with minor modification. For the trachea, the following morphological changes were included: cilia loss of mucous epithelium, extrusion of balloon-like cell and necrotic exfoliation of mucous layer, lymphocyte and heterophil infiltration in submucosal layer, hyperemia, and/or hemorrhage. For the kidney as followed: glomerular atrophy and fragmentation, tubular degeneration (granular and/or vacuolar degeneration) and necrosis (detached and broken epithelium), interstitial infiltration of lymphocytes and heterophils, hyperemia, and/or hemorrhage. The distribution and extent of the aforementioned lesions in the trachea and kidney of each chicken were scored as followed: 0, absence of lesion; 1, lesion represented in fewer than 10% of the involvement of tissue; 2, lesion represented in 10–50% involvement; 3, lesion represented in 50–90% involvement; and 4, lesion represented in more than 90% involvement. The pathological lesions of trachea and kidney were scored by 3 experienced veterinary pathologists (Dr. Guangxing Li, Ruili Zhang, and Xiaodan Huang, Northeast Agricultural University, Harbin, PR China), which were blinded to the identities of the experimental groups. The pathological injuries of trachea and kidney were quantitatively analyzed, and the statistical results were given in **Tables 2** and **3**.

### Ribonucleic Acid Extraction and Reverse Transcription

Treated cell samples were repeatedly frozen and thawed three times, and the equiponderant trachea and kidney tissues from each chick at various time points were prepared. Then, the total RNA was extracted using a Universal RNA Extraction Kit (Thermo Fisher Scientific, USA) according to the manufacturer’s instructions.

All the concentration of RNA and the A260/A280 ratio were determined with NanoDrop 2000 Spectrophotometer (Thermo Fisher Scientific, USA), and the integrity of the extracted RNA was detected by agarose gel electrophoresis. The total RNAs were reverse transcribed into cDNAs by PCR instrument (TaKaRa, Japan) for qRT-PCR, and the cDNAs were stored at −20°C.

### Quantitative Real-Time Polymerase Chain Reaction

The relative quantification analyses was used to determine the levels of mRNA expression of target genes, including N gene of IBV, IL-6, TNF-α, IL-1β, IFN-α, IFN-β, MDA5, MAVS, and NF-κB by an applied LightCycler 96 Real-Time PCR System (Roche, Switzerland). All primer pairs for real-time PCR detection are listed in **Table 1**. These primers were synthesized by GENEWIZ Biological Technology Co., Ltd. (Suzhou, China). The relative mRNA expression levels of target genes were calculated by using the 2^−ΔΔCt^ method (Yu et al., 2017).

**Table 1:** Sequences of primers used for the quantitative reverse transcription polymerase chain reaction assays.

Gene	Primer sequences (5’-3’)	Amplicon size (bp)	Annealing (°C)	Accession number
IBV-N	F: CAAGCTAGGTTTAAGCCAGGTR: TCTGAAAACCGTAGCGGATAT R: TCTGAAAACCGTAGCGGATAT	189 bp	58.3	FJ904723.1
IL-6	F: ATCCCTCCTCGCCAATCTGR: CCTCACGGTCTTCTCCATA R: CCTCACGGTCTTCTCCATA	103 bp	61.2	HM179640.1
TNF-α	F: CAGATGGGAAGGGAATGAACR: AGAGCATCAACGCAAAAGGG R: AGAGCATCAACGCAAAAGGG	268 bp	58.1	AY765397.1
IFN-α	F:GGACATGGCTCCCACACTAC R:TCCAGGATGGTGTCGTTGAAG R:TCCAGGATGGTGTCGTTGAAG	75 bp	60.0	X92476.1
IFN-β	F:GCCCACACACTCCAAAACACTGR: TTGATGCTGAGGTGAGCGTTG R: TTGATGCTGAGGTGAGCGTTG	151 bp	61.5	KF741874.1
MDA5	F:TCAGGAGGAGGACGACCACGATR: TTCCCACGACTCTCAATAACAG R: TTCCCACGACTCTCAATAACAG	168 bp	60.3	GU570144.1
MAVS	F: CCTGACTCAAACAAGGGAAGR: AATCAGAGCGATGCCAACAG R: AATCAGAGCGATGCCAACAG	123 bp	58.5	GU570144.1
NF-κB	F:TCTGAACAGCAAGTCATCCATAACGR: AAGGAAGTGAGGTTGAGGAGTCG R: AAGGAAGTGAGGTTGAGGAGTCG	250 bp	61.3	M86930.1
β-actin	F: ATTGCTGCGCTCGTTGTT R: CTTTTGCTCTGGGCTTCA	189 bp	60.0	K02173.1

The amplification system was 2 μl cDNA, 0.6 μl forward and reverse primers, 10 μl universal SYBR Green (ROX) and 6.8 μl nuclease-free water, and the final volume of each system was 20 μl. The amplification reaction of qRT-PCR assays was conducted according to the following thermal profile: pre-incubation 1 cycles at 95°C for 600 s, followed by 2 step amplification: 42 cycles at 95°C for 15 s and 60°C for 30 s, followed by melting: 1 cycles at 95°C for 10 s, 65°C for 60 s, and 97°C for 1 s, followed by cooling 1 cycles at 37°C for 30 s.

### High-Performance Liquid Chromatography/Diode Array Detector/Electrospray Ionization–Mass Spectroscopy Analysis of the *Hypericum perforatum* Ethyl Acetate

HPLC/diode array detector/ESI-MS analysis was performed on a Waters 2695 HPLC equipped with diode array detector and Waters Micromass ZQ ESI-electrospray (Waters, USA) operating in positive and negative ion mode. According to relevant reports (Seger et al., 2004; Jesionek et al., 2015), the HPLC condition was determined with a minor modification. HPE analysis was performed using an eclipse XDB-C18 column (100 mm x 4.6 mm, 5 μm, Agilent Technologies Inc. USA). The mobile phase consisted of water (A) and acetonitrile (B) at a constant flow rate (1.0 ml/min). The solvent gradient elution method was as follows: 0–5 min, 5–95% B and 5–8 min, 95% B. The detection wavelength was 0–700 nm, the column temperature was room temperature, and the injection volume was 10 μl.

The operating conditions of the mass spectrometer were dry gas temperature, 350°C, flow rate, 50 L/min; nebulizer pressure, 30 psi; sheath gas temperature, 250°C, flow rate, 10 L/min; fragmenter voltage, 100 V; capillary voltage, 3,500 V; mass range, 50 –1,100 D.

### Determination of Hyperoside, Quercitrin, Quercetin, Pseudohypericin, and Hypericin in the *Hypericum perforatum* Ethyl Acetate

The sample solution was prepared by dissolving 100.0 mg dried HPE in 25 ml mobile phase and filtered by 0.22 μm filter before HPLC analysis. The content of hyperoside, quercitrin, quercetin, pseudohypericin, and hypericin in the HPE was determined by HPLC instrument and the chromatographic conditions described above. In the 25 ml analytical solvent, the hyperoside standard of 2.5 mg, or the quercitrin standard of 5.0 mg, or the quercetin standard of 2.0 mg, or the pseudohypericin standard of 1.0 mg, or the hypericin standard of 1.0 mg are dissolved to prepared stock solution, respectively. According to the guide of International Conference on Harmonisation of Technical Requirements for Registration of Pharmaceuticals for Human Use (Singh, 2015; Armutcu et al., 2018; Hsi et al., 2019), the signal-to-noise ratio (S/N) of 3 and 10 were defined as the detection limit (LOD), and the quantitative limit (LOQ), respectively. In order to detect the S/N, the stock solution of hyperoside, quercitrin, quercetin, pseudohypericin, and hypericin were diluted to different concentrations, respectively. In order to obtain the standard curve, the stock solution of the above standard compounds was diluted into six appropriate dilution concentrations. Quantification was conducted by using a six-point standard curve and an external standard method. Intra-day and inter-day precision for hyperoside, quercitrin, quercetin, pseudohypericin, and hypericin were used to evaluate the repeatability and reproducibility of the established method.

### Statistical Analysis

The number of the experiment repetition in all the experiment was three times. The experimental data was analyzed with SPSS 17.0 software (SPSS Inc., Chicago, IL, USA). The results are expressed as the means ± standard deviation (SD). Differences between groups were evaluated using the one-way analysis of variance (ANOVA) of two tailed test. *p* < 0.05 were considered as statistically significant, and *p* < 0.01 were considered as highly significant.

## Results

### Maximum Nontoxic Concentration of *Hypericum perforatum* Ethyl Acetate, *Hypericum perforatum Water*, and SEE

**Figure 1:**
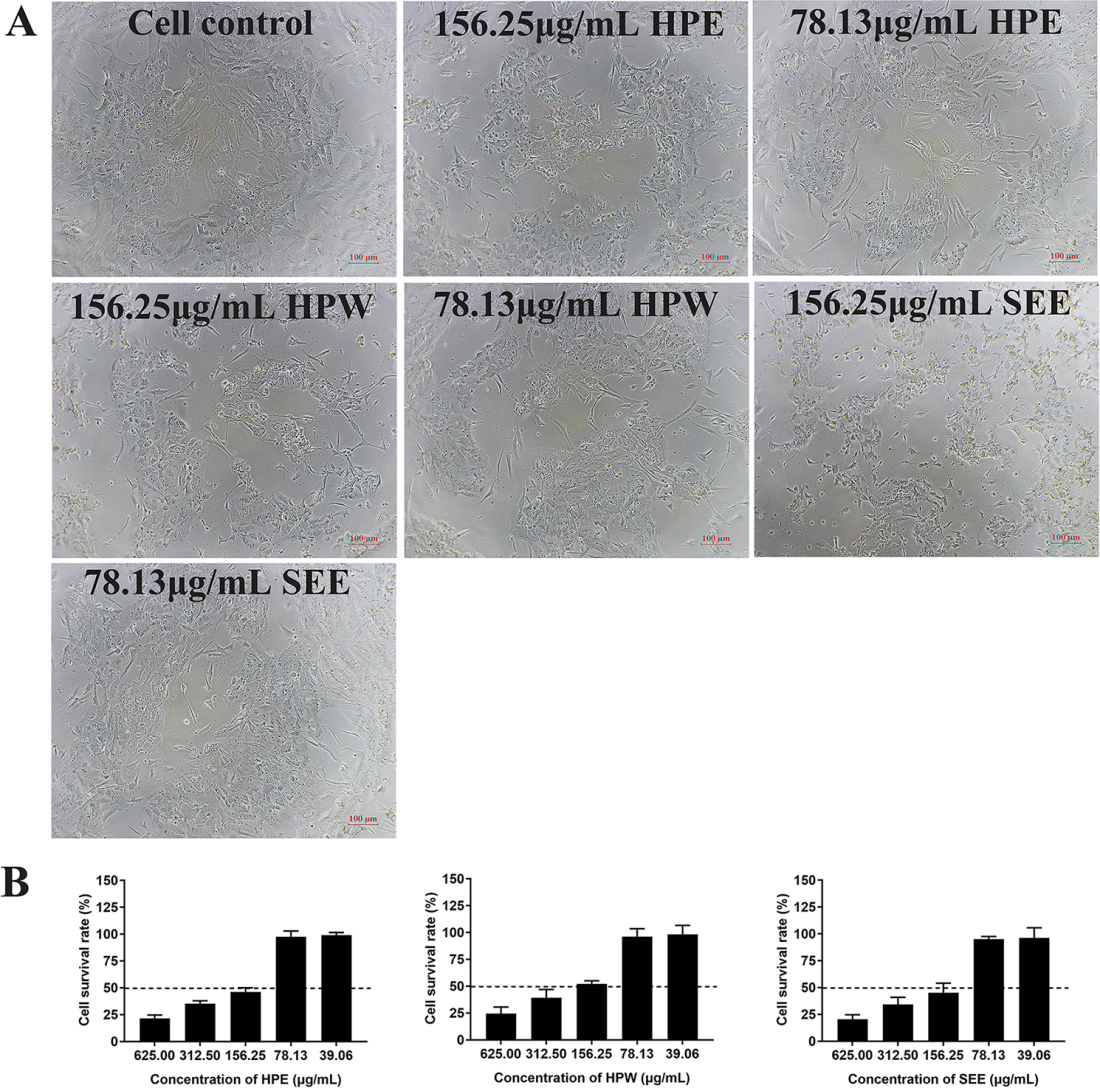
The impact of *Hypericum perforatum* ethyl acetate (HPE), *H. perforatum* water (HPW), and SEE on chicken embryo kidney cell viability. **(A)** Trypan blue staining is used to evaluate the vitality of cells. The mock cells and the cells treated with maximum non-toxic concentration of HPE, HPW, and supercritical extraction extract (SEE) were stained with trypan blue, respectively. Cells that are not stained with trypan blue are considered to be viable. **(B)** Cell viability was determined by MTT assay. The survival rate of cells treated with different concentrations of HPE, HPW, and SEE was given. More than 50% cell survival rate was considered to be the maximum non-toxic concentration of SEE, HPE, and HPW. Data are expressed as mean ± SD of three independent experiments.

The cell viability after trypan blue staining was observed under optical microscope and further confirmed by MTT method. The results showed that the maximum nontoxic concentration of HPE, HPW, and SEE was 78.13 μg/ml. When the concentration of HPE, HPW, and SEE was lower than 78.13 μg/ml, trypan blue staining showed cell was survival (**Figure 1A**). At the same time, the cell survival rate measured by MTT method was close to 100%, which was further explained that 78 μg/ml of the drug had no significant effect on the cells (**Figure 1B**).

### Adaptation and Replication of Infectious Bronchitis Virus in Chicken Embryo Kidney Cells

**Figure 2:**
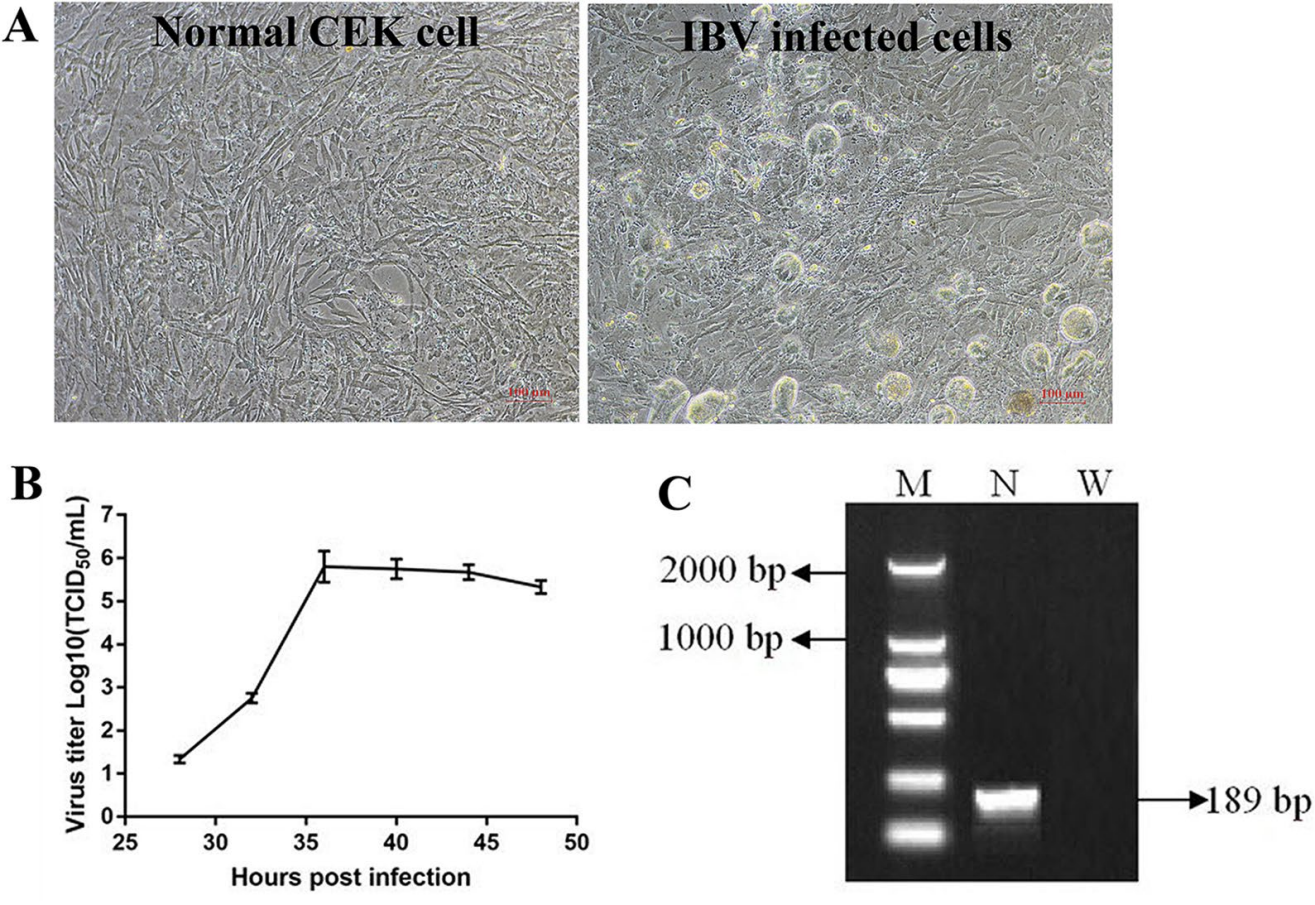
Adaptation and replication of infectious bronchitis virus (IBV) in chicken embryo kidney (CEK) cells. **(A)** IBV adapts to CEK cells and produces stable typical cytopathic effect. **(B)** Virus growth curve of IBV in CEK cells after IBV adaptation to cells. **(C)** The results of reverse transcription polymerase chain reaction identification of IBV M41 in CEK cells. In which, M stands for deoxyribonucleic acid marker, N stands for polymerase chain reaction product for IBV N gene, W stands for negative water control.

When IBV was propagated in CEK cells to the tenth generation, stable typical cytopathic effect (CPE) appeared at 36 h after IBV infection (**Figure 2A**). At the beginning, cells infected with IBV became round and refractive, then some cell exfoliated from the flask and left empty hole, some cells fused together and became multinuclear giant cells, indicating that IBV adapted to CEK cells. The results of virus growth curve showed that IBV could infect and replicate in CEK cells and reached the highest titer of 10^−5.8^ TCID_50_ per 100 μl at 36 h post-inoculation (**Figure 2B**). At the same time, the existence of IBV M41 was identified by RT-PCR (**Figure 2C**).

### Antiviral Effect of Supercritical Extraction Extract, *Hypericum perforatum* Ethyl Acetate, and *Hypericum perforatum Water In Vitro*

**Figure 3:**
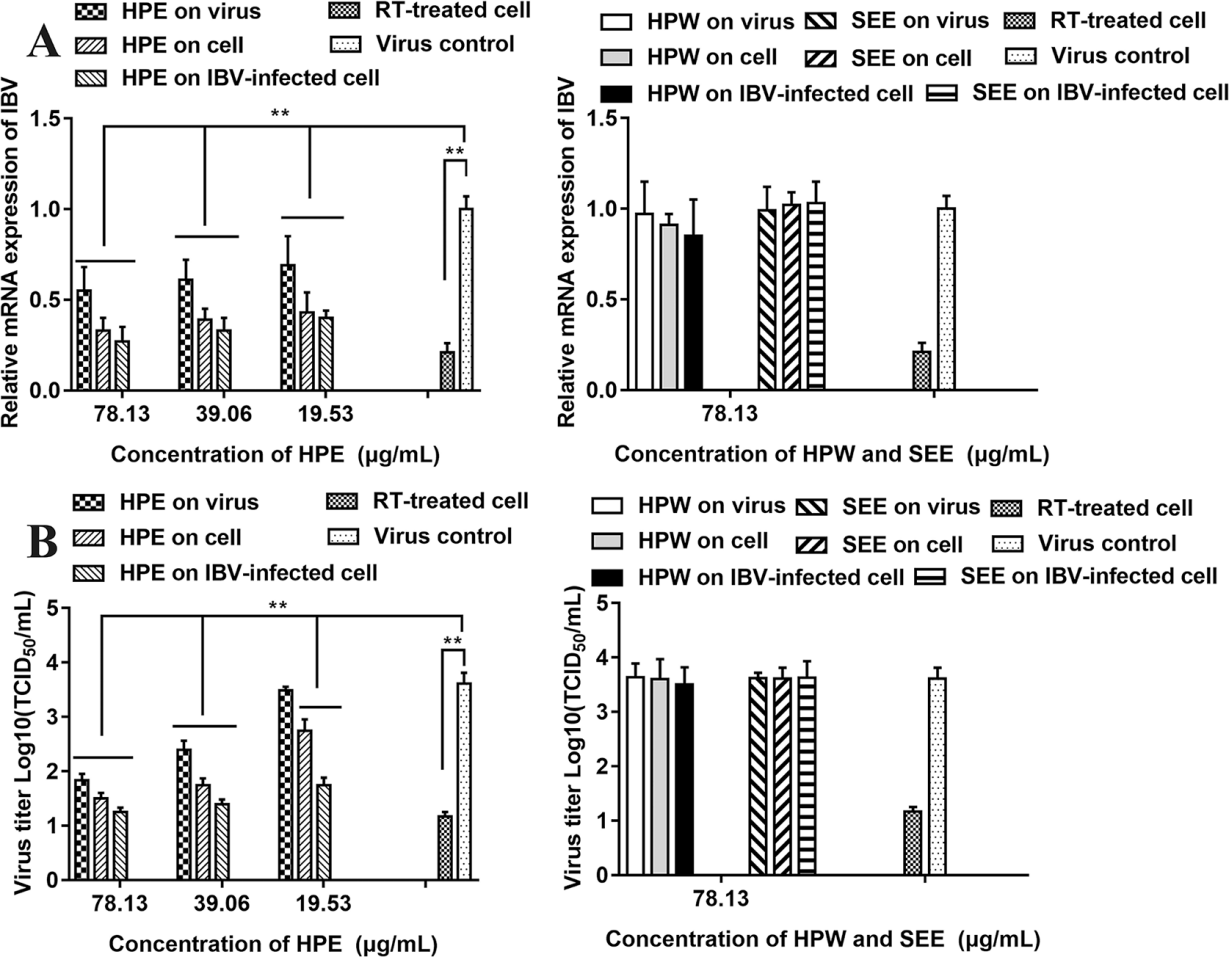
The effect of *Hypericum perforatum* ethyl acetate (HPE), *H. perforatum* water (HPW), and supercritical extraction extract (SEE) on the relative messenger ribonucleic acid (mRNA) expression levels and virus titer of infectious bronchitis virus (IBV) in chicken embryo kidneys. To investigate the impact of HPE, HPW, and SEE on cell, IBV-infected cells, and on the virus, the relative mRNA expression levels of IBV-N gene **(A)** and the virus titer **(B)** were detected, respectively. Viral infection cell and 10 μg/ml ribavirin (RT) treated cell were included as controls. Data are expressed as mean ± SD of three independent experiments (t-test, **p < 0.01).

The relative mRNA expression level of IBV-N gene was detected by qRT-PCR and the virus titer of IBV was determined by TCID_50_ to analyze the antiviral effect of HPE, HPW and SEE (**Figure 3**). It could be seen from **Figure 3** that under the maximum non-toxic concentration of the drug, the inhibition of HPE on IBV was significantly greater than that of HPW and SEE. HPW had a very weak effect on IBV and SEE had no effect on IBV. Therefore, it was determined that the antiviral part of *H. perforatum* extract was ethyl acetate layer.

As can be seen from **Figure 3A**, in the three experimental designs, with the increase of HPE concentration, the mRNA level of IBV decreased significantly in a dose-dependent manner. In addition, at the same drug concentration, the relative expression level of virus mRNA was the lowest when HPE directly treated the infected virus cells, followed by HPE pre-treated cells before infection, and then HPE pre-treated virus before infection.

It can be known from the **Figure 3B**, with the increase of HPE concentration, the titer of IBV decreased gradually in a dose-dependent manner. While, at the same drug concentration, the virus titer was the lowest when HPE directly treated the infected virus cells, followed by HPE pre-treated cells before infection, and followed by HPE pre-treated virus before infection. At the same time, the antiviral impact of HPE directly treated the IBV-infected cells at the concentration of 78.13 µg/ml, was similar to RT at the concentration of 10 µg/ml. Therefore, HPE was selected for subsequent experiments *in vivo* and *in vitro*, furthermore the HPE directly treated the IBV-infected cells was selected *in vitro* experiments.

### IFA Analysis Confirmed Inhibitory Effect of *Hypericum perforatum* Ethyl Acetate

**Figure 4:**
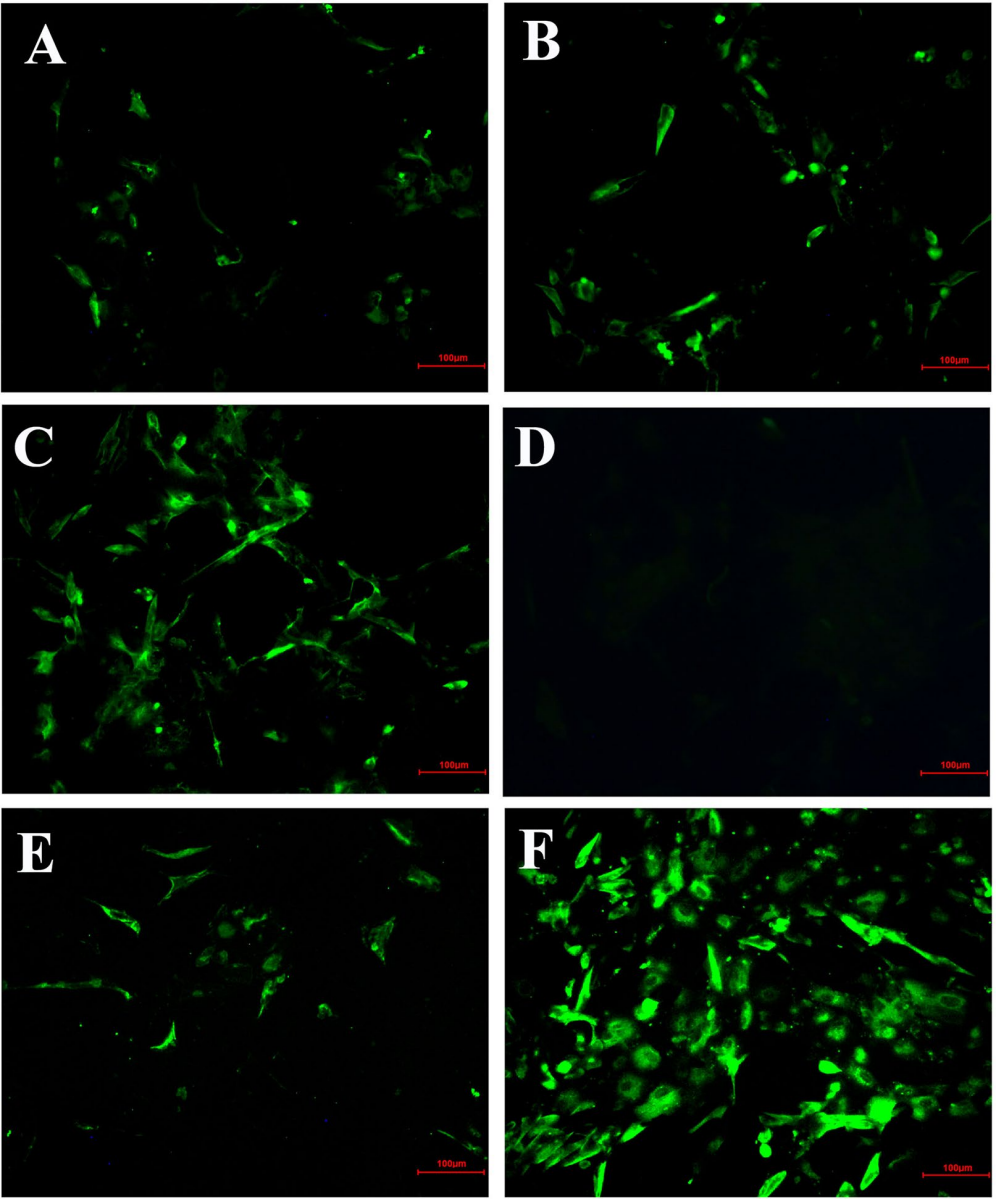
The inhibitory effects of *Hypericum perforatum* ethyl acetate (HPE) on infectious bronchitis virus (IBV) by immunofluorescence assays (IFA). The chicken embryo kidney (CEK) cells were infected with 100 TCID_50_ IBV for 2 h at 37°C followed by incubation with HPE at the concentration of 78.13 µg/ml **(A)**, 39.06 µg/ml **(B)**, and 19.53 µg/ml **(C)** for 30 h. The CEK cells were cultured in M199 as negative control **(D)**, and treated with ribavirin (10 μg/ml) as reference **(E)**, and incubated with 100 TCID_50_ IBV as positive control **(F)**, respectively. The fluorescence intensity (20 ×) produced by IBV on CEK cells was photographed.

In order to further confirm the inhibitory effect of HPE on IBV-infected cells, the fluorescent signal of virus was detected by IFA (**Figure 4**). From the **Figure 4**, it can be seen that the CEK cells infected with IBV produced a strong fluorescence signal at 30 h after infection. On the contrary, the fluorescence signal of CEK cells infected with IBV and treated with HPE was weakened, and the fluorescence signal decreased in a dose-dependent manner with the increase of HPE concentration. This further confirmed that HPE has a very good inhibitory effect on IBV infected cells.

### Effect of *Hypericum perforatum* Ethyl Acetate on the Messenger Ribonucleic Acid Expression Level of Gene *In Vitro*

**Figure 5:**
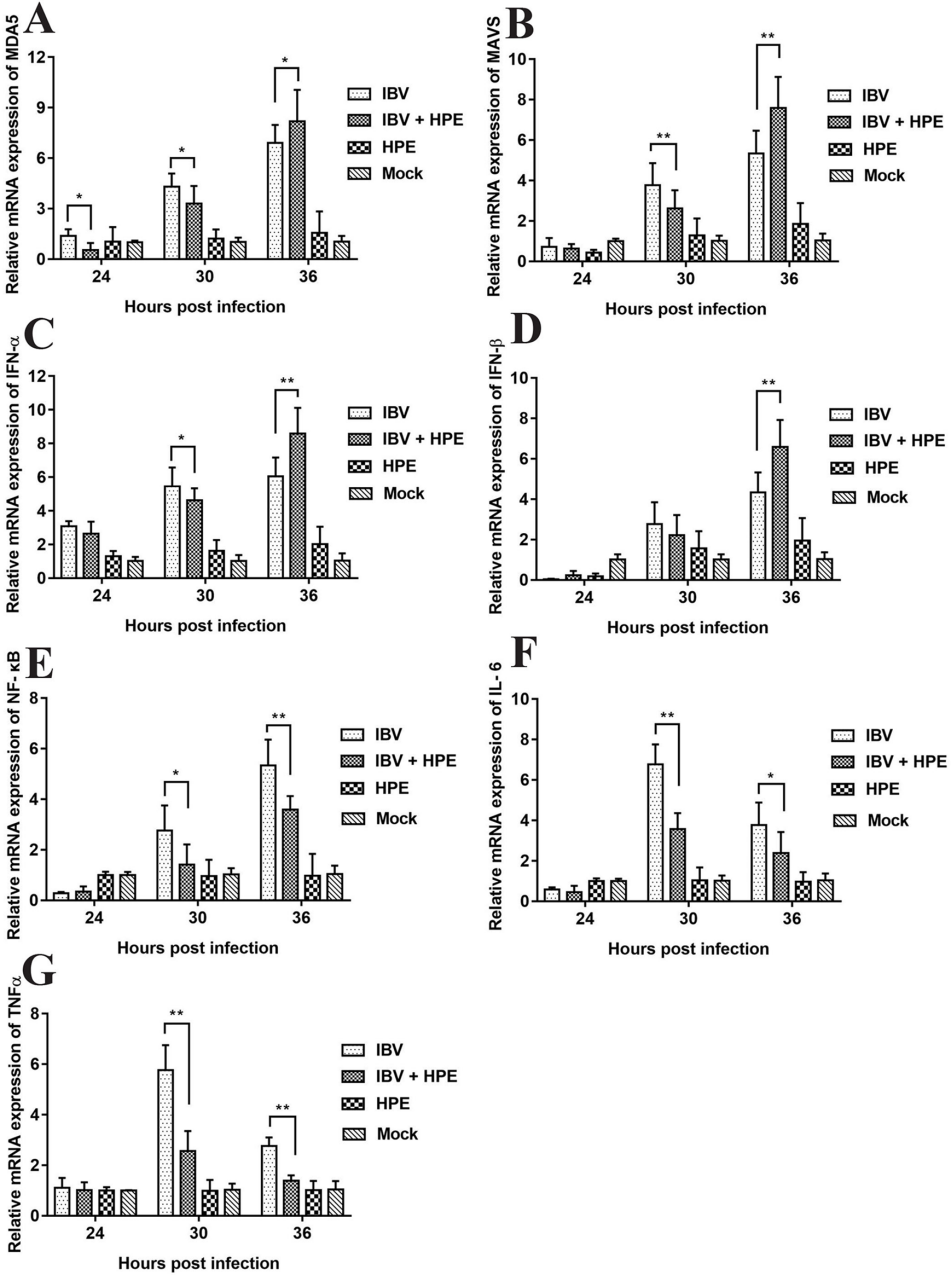
The effects of *Hypericum perforatum* ethyl acetate on the messenger ribonucleic acid (mRNA) expression level of related genes *in vitro*. Chicken embryo kidney (CEK) cells were infected with 100 TCID_50_ infectious bronchitis virus (IBV) at 37°C for 2 h, and then incubated with 78.13 μg/ml HPE. The CEK cells were treated with M199, 100 TCID_50_ IBV, and 78.13 µg/ml HPE as the control, respectively. Subsequently, total RNA was extracted from CEK cell samples at 24, 30, and 36 h after treatment. The relative mRNA expression of melanoma differentiation-associated protein 5 **(A)**, mitochondrial antiviral signaling gene **(B)**, interferon alpha **(C)**, interferon beta **(D)**, nuclear factor kappa beta **(E)**, IL-6 **(F)**, and tumor necrosis factor alpha **(G)** were determined by quantitative reverse transcription polymerase chain reaction. The differences between means were considered significant at *p < 0.05 and highly significant at **p < 0.01 when compared with the IBV-infected cell.

In order to study the effect of HPE on gene mRNA expression induced by IBV infection in CEK cells, the mRNA expression level of related genes were determined, including MDA5, MAVS, IFN-α, IFN-β, NF-κB, IL-6, and TNF-α. As can be seen from **Figure 5**, HPE treatment significantly affected the mRNA levels of MDA5, MAVS, IFN-α, and IFN-β after IBV infection at 30 and 36 h, and the mRNA expression levels of these genes changed similarly. These data suggested that HPE could increase mRNA expression level of type I interferon in the late stages of IBV infection, which is possibly related to MDA5 signaling pathway. In addition, HPE treatment significantly reduced the mRNA expression levels of NF-κB, IL-6, and TNF-α, which were up-regulated after IBV infection at 30 and 36 h. These data demonstrated that HPE could decrease the mRNA expression of pro-inflammatory genes, which is possibly related to NF-κB signaling pathway.

### Antiviral Effect of *Hypericum perforatum* Ethyl Acetate *In Vivo*

#### Effect of *Hypericum perforatum* Ethyl Acetate on Pathological Injury of Trachea and Kidney Caused by Infectious Bronchitis Virus

**Figure 6:**
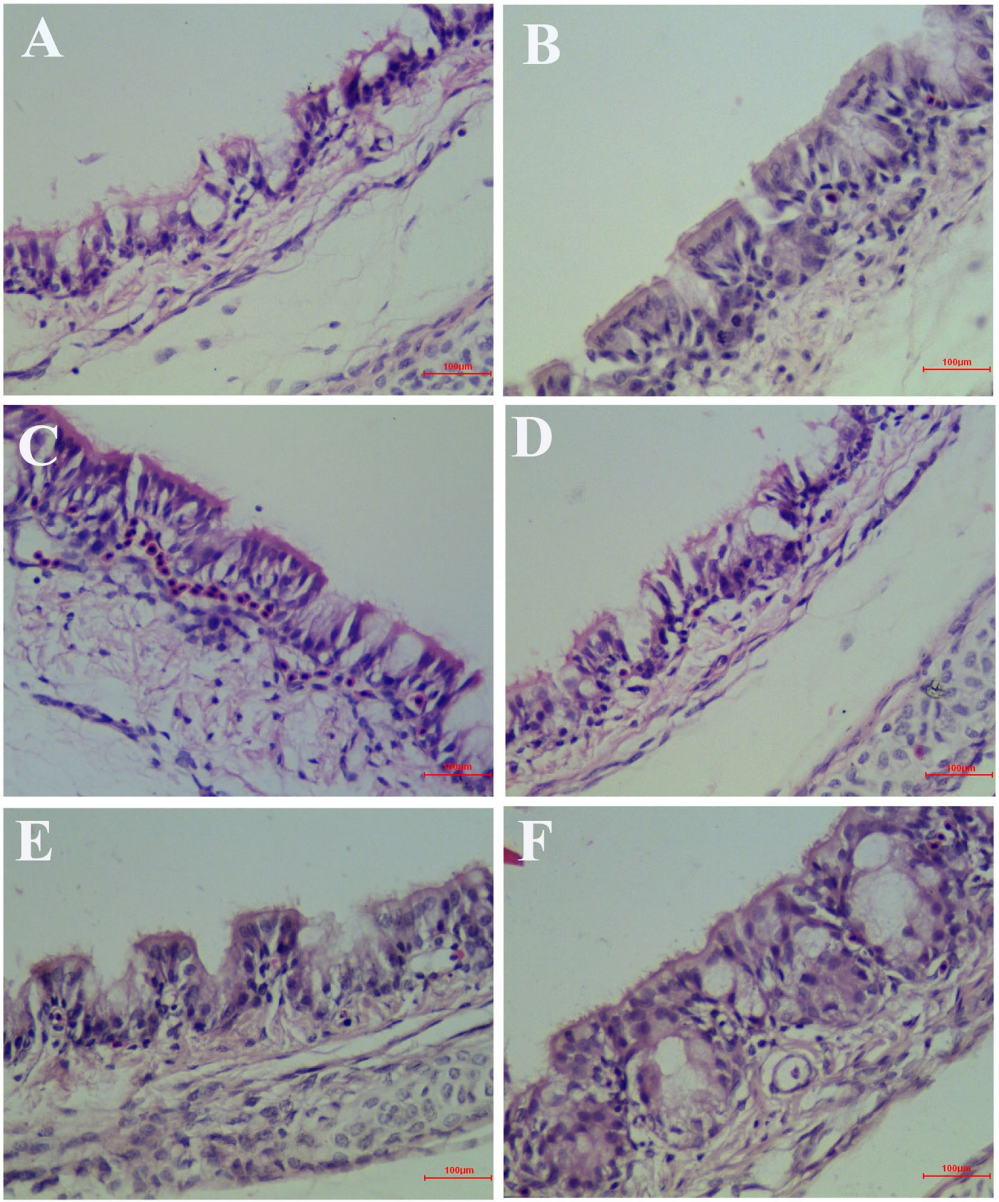
Inhibitory effects of *Hypericum perforatum* ethyl acetate (HPE) on infectious bronchitis virus (IBV)-induced tracheal injury. Trachea from each experimental group was processed for histological evaluation at 6 day after infection. **(A)** Trachea section from the IBV-infected group. **(B)** Trachea section from the control group. **(C)** Trachea section from IBV-infected and treated with ribavirin (60 mg/kg). **(D**–**F)** The trachea section from the IBV-infected and treated with different concentrations of HPE at 120, 240, and 480 mg/kg, respectively. Representative histological tissue sections were stained with hematoxylin and eosin (H.E.).

At 6 days after infection, the symptoms that appeared in the IBV infected group were sneezing, tracheal wet rales, mouth breathing, coughing, ruffled feathers, and frequently shaking. But the symptoms that appeared in the HPE and RT treatment group such as sneezing, tracheal wet rales, mouth breathing, coughing, feather wrinkling, and frequent trembling were mild. And the trachea and kidneys of different groups of SPF chickens were conducted for histopathology assay. As can be seen from **Figure 6**, most of the tracheal cilia in the IBV infected group fell off, the mucosal epithelial cells exfoliated and disappeared, the submucosal structure was loosely arranged, and there was a certain amount of serous exudation, inflammatory cells, and red blood cells. In the HPE treatment group, with the gradual increase of the dosage, the shedding of tracheal cilia and epithelial cells was gradually decreased, there were a small number of inflammatory cells and erythrocytes, and the exudation of inflammatory serous fluid decreased.

**Figure 7:**
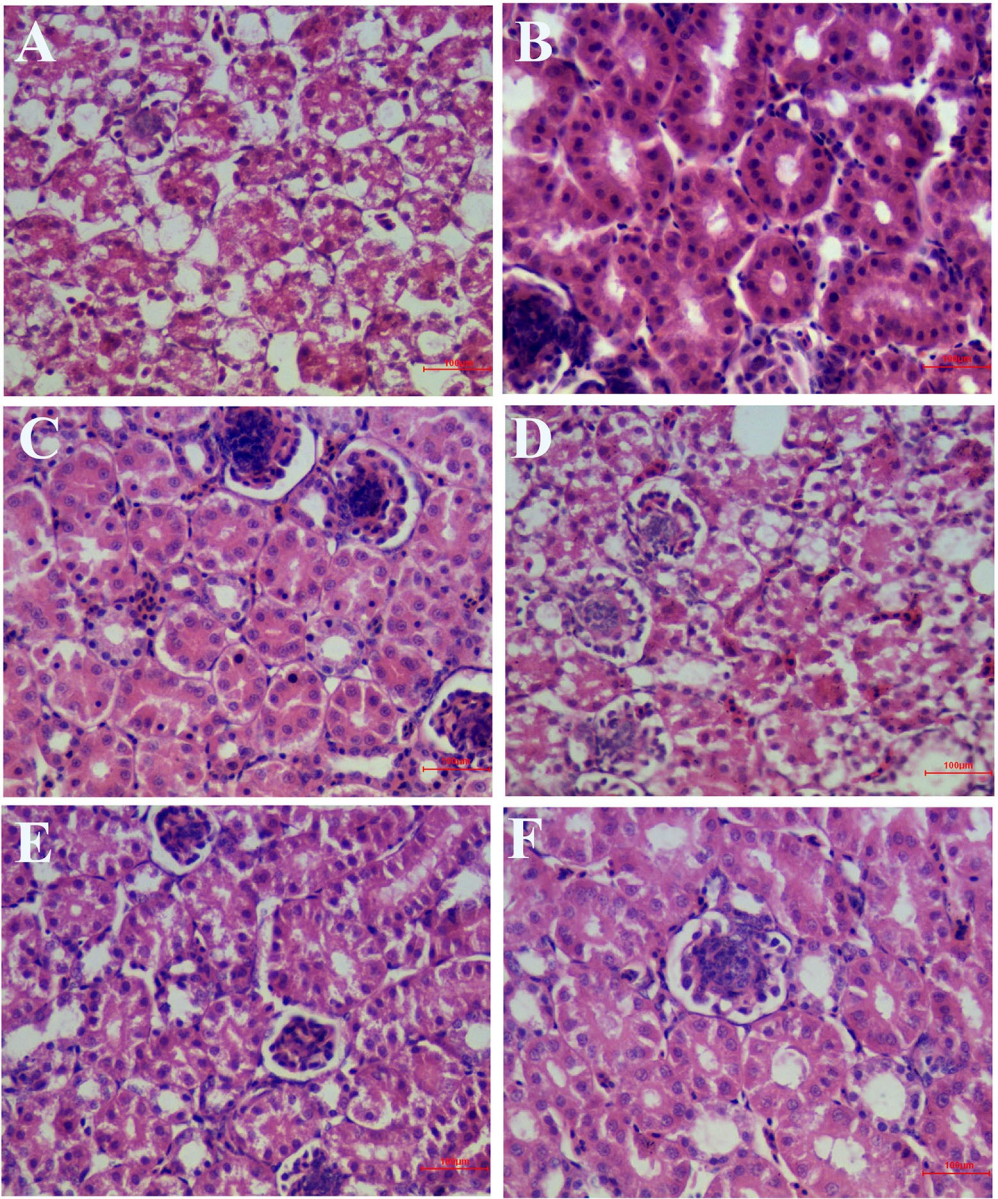
Inhibitory effects of *Hypericum perforatum* ethyl acetate (HPE) on infectious bronchitis virus (IBV)-induced kidney injury. Kidney from each experimental group was processed for histological evaluation at 6 day after infection. **(A)** Kidney section from the IBV-infected group. **(B)** Kidney section from the normal control group. **(C)** Kidney section from IBV-infected and treated with ribavirin (60 mg/kg). **(D–F)** Kidney section from the IBV-infected and treated with different concentrations of HPE at 120, 240, and 480 mg/kg, respectively. Representative histological tissue sections were stained with H.E.

As shown in **Figure 7**, in the IBV infected group, a large number of renal tubular epithelial cells were degenerated and necrotic, the nucleus were concentrated, fragmented and dissolved, the renal tubular epithelial cells fell off into the lumen, dissolved or disappeared, the structure of renal tubules was incomplete, and there were a certain number of inflammatory cells and red blood cells in the interstitium. The wall of renal capsule was ruptured and a certain amount of inflammatory cells and serous exudation could be seen in the lumen of renal capsule. In the HPE treatment group, with the increase of the dosage, the exfoliation and dissolution of renal tubular epithelial cells decreased gradually, the structure of renal tubule was gradually complete, the number of red blood cells and inflammatory cells in the stroma was less, and the structure of renal capsule wall was more complete. There was little infiltration of inflammatory cells in the renal vesicle cavity.

As can be seen from **Tables 2** and **3**, the pathological scores of trachea and kidney in IBV group were highly significantly higher than those in NC group (*p* < 0.01). Compared with the IBV group, the pathological scores of trachea and kidney in the HPE-TH group and the HPE-TM group decreased highly significantly (*p* < 0.01), and the score was similar to that of the RT group. At the same time, with the increase of HPE dose, the score decreased in a dose-dependent manner. This indicated that HPE had better anti-IBV effect.

**Table 2:** Histopathologic scoring of trachea.

Group	n	Cilia loss	Degeneration and necrosis of epithelium	Infiltration in submucosal layer	Hyperemia and/or hemorrhage	Total score
IBV	6	3.9 ± 0.3	3.9 ± 0.2	1.8 ± 0.3	0.8 ± 0.2	10.4 ± 0.6
NC	6	0.1 ± 0.1	0.1 ± 0.1	1.2 ± 0.2	0.8 ± 0.2	2.2 ± 0.2**
RT	6	1.9 ± 0.2	0.8 ± 0.2	1.6 ± 0.3	1.7 ± 0.3	6.0 ± 0.4**
HPE-TL	6	3.2 ± 0.3	3.1 ± 0.2	2.7 ± 0.3	0.8 ± 0.3	9.8 ± 0.5
HPE-TM	6	1.7 ± 0.2	1.8 ± 0.3	1.5 ± 0.3	0.8 ± 0.2	5.8 ± 0.4**
HPE-TH	6	1.6 ± 0.3	0.7 ± 0.3	0.9 ± 0.2	0.7 ± 0.3	3.9 ± 0.4**

The difference was considered highly significant at **p < 0.01 vs. IBV infection group.

**Table 3:** Histopathologic scoring of kidney.

Group	n	Glomerular atrophy and fragmentation	Tubular degeneration and necrosis	Interstitial infiltration	Hyperemia and/or hemorrhage	Total score
IBV	6	3.7 ± 0.2	3.9 ± 0.1	2.5 ± 0.4	1.7 ± 0.2	11.8 ± 0.5
NC	6	0.6 ± 0.1	0.6 ± 0.1	0.9 ± 0.2	0.1 ± 0.1	2.2 ± 0.2**
RT	6	0.9 ± 0.1	1.4 ± 0.2	1.4 ± 0.3	0.6 ± 0.2	4.3 ± 0.3**
HPE-TL	6	3.4 ± 0.3	3.8 ± 0.3	2.3 ± 0.4	1.6 ± 0.3	11.1 ± 0.6
HPE-TM	6	1.2 ± 0.2	2.0 ± 0.4	1.5 ± 0.4	0.8 ± 0.2	5.5 ± 0.5**
HPE-TH	6	0.8 ± 0.2	1.2 ± 0.4	1.0 ± 0.1	0.7 ± 0.2	3.7 ± 0.4**

The difference was considered highly significant at **p < 0.01 vs. IBV infection group.

### The Effect of *Hypericum perforatum* Ethyl Acetate on the Relative Messenger Ribonucleic Acid Expression of IBV in Trachea and Kidney

**Figure 8:**
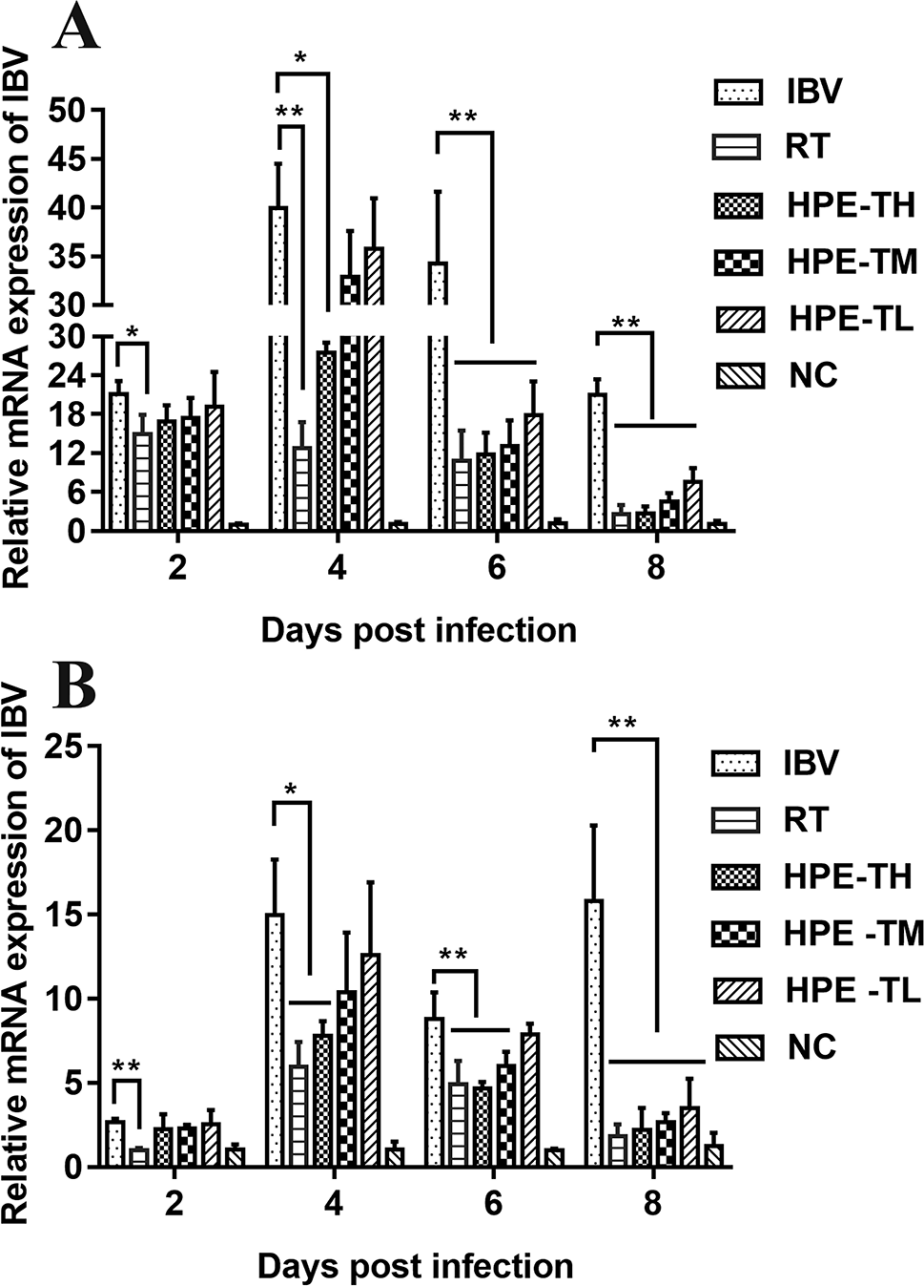
The effects of *Hypericum perforatum* ethyl acetate (HPE) on infectious bronchitis virus (IBV) messenger ribonucleic acid (mRNA) expression levels of trachea **(A)** and kidney **(B)**. SPF chickens were infected with IBV, followed by oral administration of HPE and ribavirin, respectively. Total RNA were subsequently extracted from trachea and kidney at 2, 4, 6, and 8 day post-infection. The relative mRNA expression of IBV was determined by quantitative reverse transcription polymerase chain reaction. The differences between means were considered significant at *p < 0.05 and highly significant at **p < 0.01.

To confirm the inhibitory effect of HPE, IBV mRNA levels in trachea and kidney of different groups were measured by real-time qRT-PCR. As shown in **Figure 8**, Compared with NC group, the expression level of mRNA in trachea and kidney of SPF chicks infected with IBV increased significantly. Compared with IBV group, the expression level of mRNA in trachea and kidney of SPF chicks treated with HPE decreased significantly. With the decrease of HPE concentration, the expression level of mRNA increased in a dose-dependent manner. With the increase of the days of IBV infection, the effect of HPE-TH and HPE-TM group on the level of mRNA expression was similar to that of RT group. These data indicated that infected chickens treated with HPE exhibited an overall reduction in viral mRNA levels.

### Effect of *Hypericum perforatum* Ethyl Acetate on the Messenger Ribonucleic Acid Expression Level of Gene in the Trachea and Kidney

**Figure 9:**
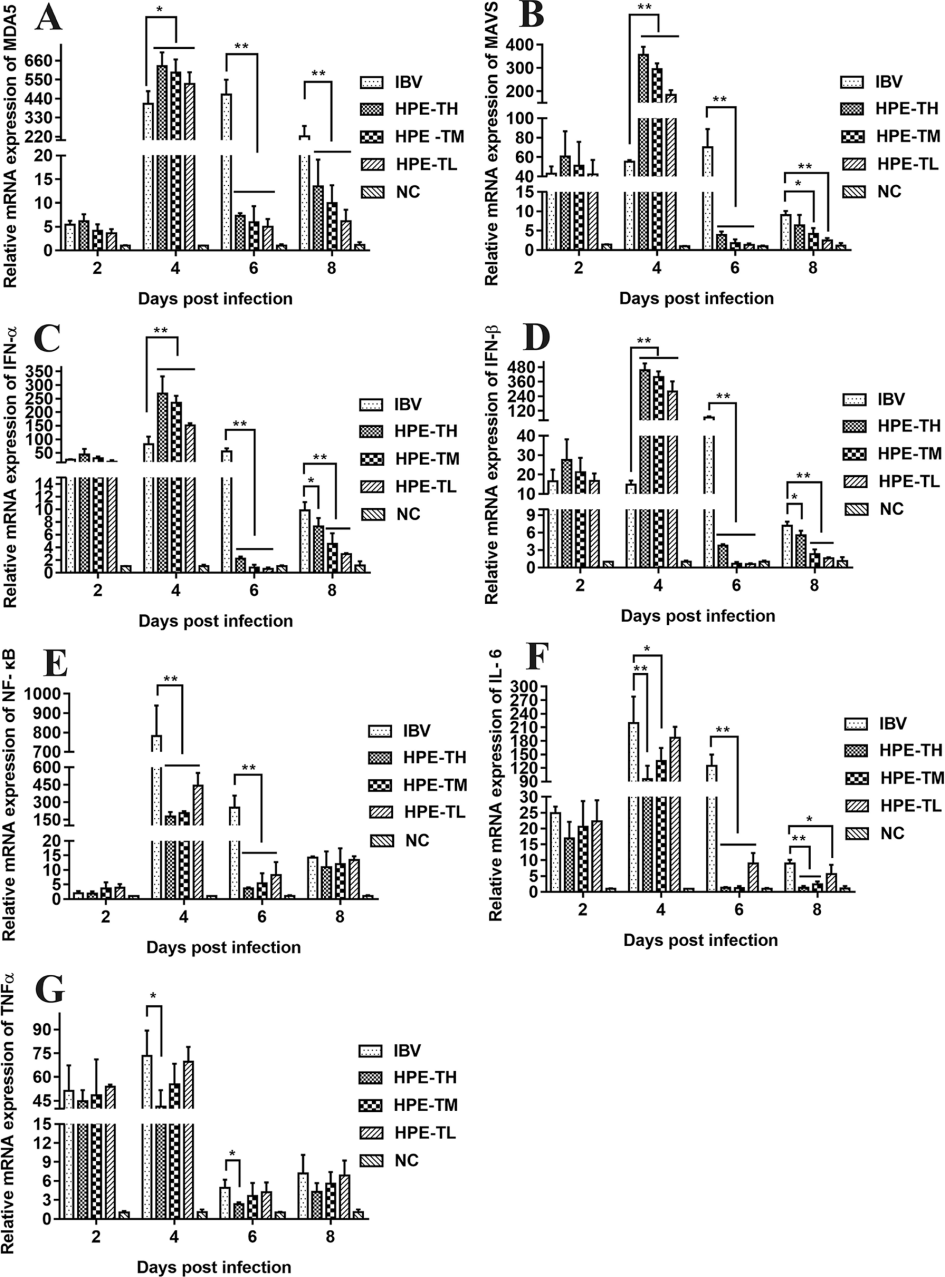
Effect of *Hypericum perforatum* ethyl acetate (HPE) on the messenger ribonucleic acid (mRNA) expression of genes in trachea. SPF chickens were infected with IBV, followed by oral administration of HPE and ribavirin, respectively. Total RNA were subsequently extracted from trachea at 2, 4, 6, and 8 day post-infection. The relative mRNA expression of **(A)** melanoma differentiation-associated protein 5, **(B)** mitochondrial antiviral signaling gene, **(C)** interferon alpha, **(D)** interferon beta, **(E)** nuclear factor kappa beta, **(F)** IL-6, and **(G)** tumor necrosis factor alpha were determined by quantitative reverse transcription polymerase chain reaction. The differences between means were considered significant at *p < 0.05 and highly significant at **p < 0.01.

**Figure 10:**
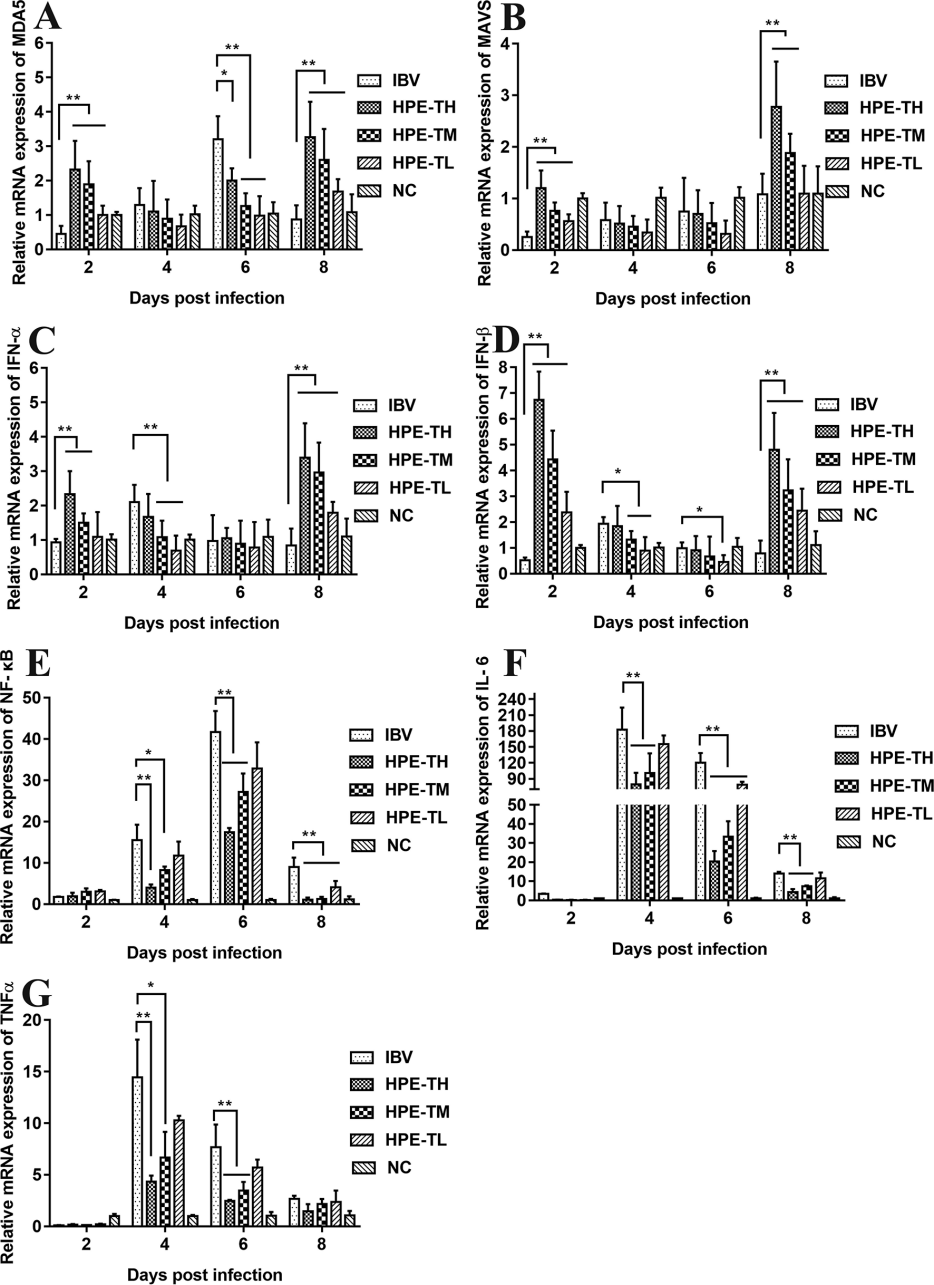
Effect of *Hypericum perforatum* ethyl acetate (HPE) on the messenger ribonucleic acid (mRNA) expression of gene in kidney. Specific pathogen-free (SPF) chickens were infected with infectious bronchitis virus (IBV), followed by oral administration of HPE and ribavirin (RT), respectively. Total RNA were subsequently extracted from kidney at 2, 4, 6, and 8 day post-infection. The relative mRNA expression of **(A)** melanoma differentiation-associated protein 5, **(B)** mitochondrial antiviral signaling gene, **(C)** interferon alpha, **(D)** interferon beta, **(E)** nuclear factor kappa beta, **(F)** IL-6, and **(G)** tumor necrosis factor alpha were determined by quantitative reverse transcription polymerase chain reaction. The differences between means were considered significant at *p < 0.05 and highly significant at **p < 0.01.

It is well known that type I interferon, including IFN-α and IFN-β, plays a very important role in antiviral activity. As can be seen from **Figures 9** and **10**, the relative mRNA expression of IL-6, TNF-α, IFN-α, IFN-β, MDA5, MAVS, and NF-κB in the trachea and kidney were up-regulated after IBV infection, which was consistent with previous studies (He et al., 2016; Chhabra et al., 2018). In HPE treatment group, the mRNA expression levels of IFN-α and IFN-β in the trachea were up-regulated in the early stage of IBV infection, and the changes of IFN-α and IFN-β were consistent with the mRNA expression of MDA5 and MAVS in the trachea. Meanwhile, in the HPE-treated group, the mRNA expression levels of IFN-α and IFN-β in the kidney were up-regulated in the late stage of IBV infection. In addition, the changes of IFN-α and IFN-β were similar to the mRNA expression levels of MDA5 and MAVS in the kidney. These data suggested that HPE may up-regulate mRNA expression levels of IFN-α and IFN-β in trachea and kidney *via* the MDA5 signaling pathway.

At the same time, it is well known that inflammatory factors play an important role in inflammation. It also plays a very important role in IBV infection. As can be seen from **Figures 8** and **9**, in the HPE-treated group, the mRNA expression levels of IL-6 and TNF-α in the trachea and kidney were down-regulated, and the changes were consistent to NF-κB mRNA expression levels, indicating that HPE may be down-regulated the mRNA expression levels of IL-6 and TNF-α by NF-κB signaling pathway. Therefore, HPE may exert an anti-IBV effect by increasing the mRNA expression levels of type I interferon and reducing mRNA expression levels of pro-inflammatory factors IL-6 and TNF-α, and this may be related to the MDA5 signal pathway and NF-κB signal pathway.

### High-Performance Liquid Chromatography/Electrospray Ionization–Mass Spectroscopy Analysis of the *Hypericum perforatum* Ethyl Acetate

**Figure 11:**
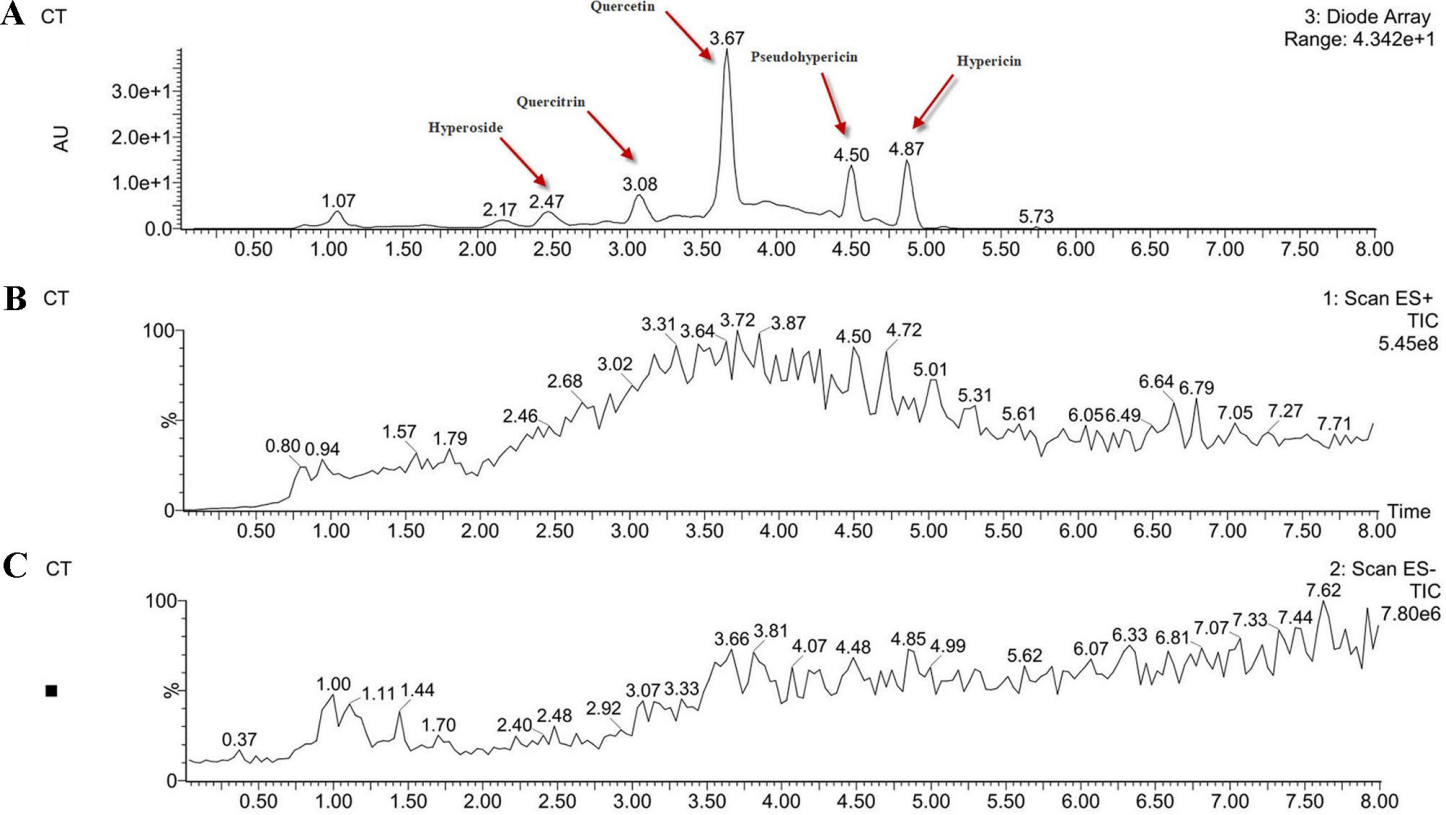
Analysis of the components of *Hypericum perforatum* ethyl acetate. **(A)** high-performance liquid chromatography chromatogram and **(B)** total ion chromatogram of the mass spectrometer in negative ion mode and **(C)** positive ion mode.

**Figure 12:**
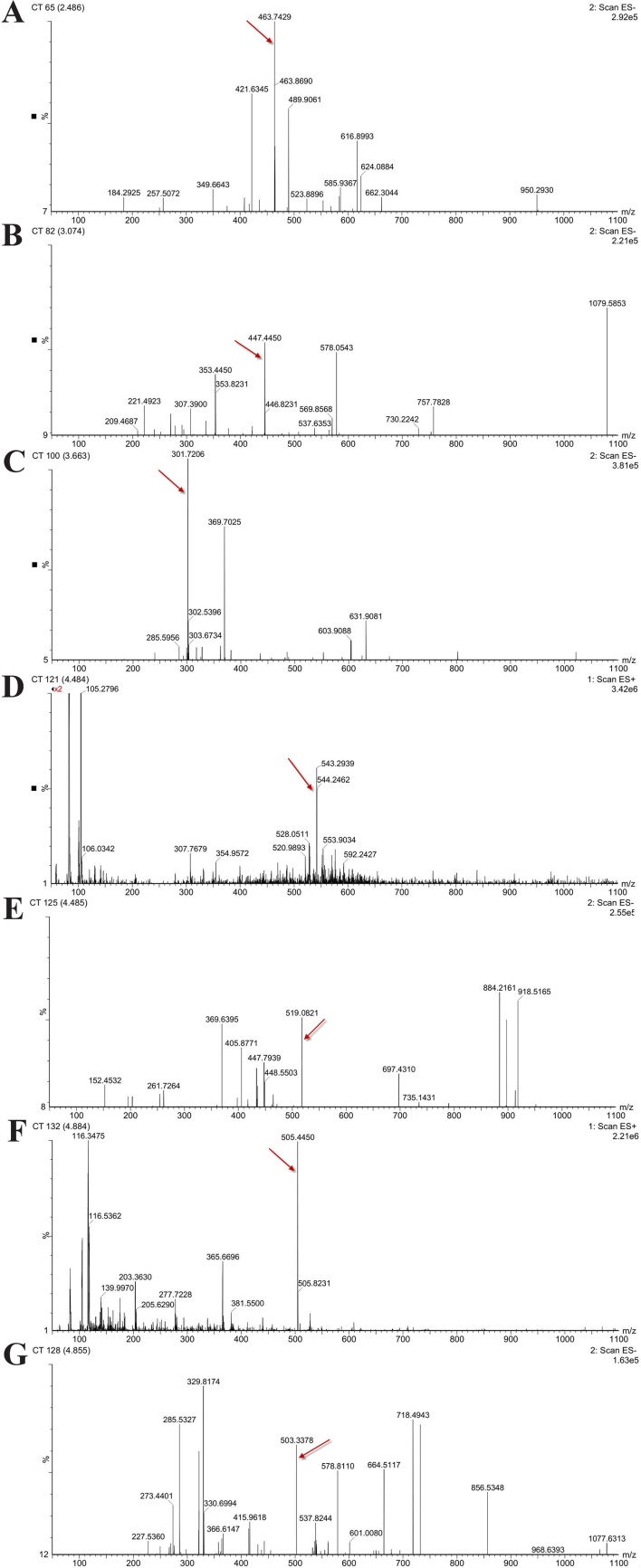
Mass spectra of five representative compounds in *Hypericum perforatum* ethyl acetate. **(A)** hyperoside, **(B)** quercitrin, **(C)** quercetin, **(D, E)** pseudohypericin, and **(F, G)** hypericin.

In order to determine the structure of the main components in the HPE, the samples were analyzed by positive and negative modes using HPLC/ESI-MS. All tested components showed their HPLC chromatogram and quasi-molecular ions [(M-H)^+^ and (M-H)^−^] (**Figure 11**). The mass spectra of these compounds were carefully examined (**Figure 12**) and compared with the standard and reference data (Seger et al., 2004;Cao et al., 2011) and found to have five peaks in the HPE (**Table 4**). These peaks corresponded to hyperoside (peak 1), quercitrin (peak 2), quercetin (peak 3), pseudohypericin (peak 4), and hypericin (peak 5).

**Table 4:** High-performance liquid chromatography/electrospray ionization–mass spectroscopy fragmentation (negative and positive ion mode) and ultraviolet–visible spectroscopy absorption data of the compounds detected in the *Hypericum perforatum* ethyl acetate.

Compound name	R_t_ (min)	UV (nm)	Negative ion mode	Positive ion mode	Molecular weight
Hyperoside	2.47	360	463.74 [M–H]^−^		464
Quercitrin	3.08	367	447.44 [M–H]^−^		448
Quercetin	3.67	373	301.72 [M–H]^−^		302
Pseudohypericin	4.50	590	519.08 [M–H]−	543.29 [M+Na]^+^	520
Hypericin	4.87	590	503.33 [M–H]−	505.44 [M+H]^+^	504

### Determination of Hyperoside, Quercitrin, Quercetin, Pseudohypericin, and Hypericin in the *Hypericum perforatum* Ethyl Acetate

The calibration curves of hyperoside, quercitrin, quercetin, pseudohypericin, and hypericin were as followed: y = 20,579x + 25,971, y = 18,877x + 41,654, y = 250,933x−272,745, y = 76,343x + 16,472, and y = 73,815x + 3,490.7, where y is peak area, x is sample concentration, and the concentration ranges were 10–60, 40–140, 10–60, 1–6, and 6–36 μg/ml, respectively. And the calibration curve was in accordance with a linear regression (*R^2^* = 0.9993–0.9997). The LOD (S/N = 3) of hyperoside, quercitrin, quercetin, pseudohypericin, and hypericin was in the range of 0.64 to 0.78 μg/ml. The LOQ (S/N = 10) of hyperoside, quercitrin, quercetin, pseudohypericin, and hypericin was in the range of 0.16 to 0.22 μg/ml. By measuring the peak area and retention time of standard compounds to evaluate the intra-day and inter-day precision, their relative SD was less than 1%. The results showed that the HPE contained 0.92% hyperoside, 1.37% quercitrin, 10.16% quercetin, 2.55% pseudohypericin, and 3.46% hypericin.

## Discussion

IBV has a variety of known strains, these strains continue to mutate, and more mutants continue to recombine, resulting in more diverse and complex genotypes and serotypes of IBV (Bande et al., 2015; Feng et al., 2015; Laconi et al., 2019). Due to the poor cross-protection of vaccines between different serotypes, it is difficult to prevent and control IBV infection. Therefore, development of an effective antiviral therapy is a crucial strategy for treating IBV infection. In this study, the anti-IBV effect of the drug was studied by three kinds of methods, qRT-PCR, determination of TCID_50_, and indirect immunofluorescence (IFA). qRT-PCR is a method of measuring the total amount of products after each reaction cycle with fluorescent chemicals in DNA amplification. IFA is a method to detect the presence of specific antigens in cells after the cells were treated with fluorescent-antibodies. TCID_50_ is the amount of infection in which 50% of the cells are infected by the virus. The anti-virus effect of drugs is often detected by qRT-PCR (Schnepf et al., 2009; Li et al., 2011; Zhang et al., 2018), IFA (Xu et al., 2008; Zhang et al., 2017a) and TCID_50_ (Xu et al., 2008; Zhang et al., 2013) in antiviral research. And these antiviral drug reports are similar to our research, so we also used these methods to detect the anti-IBV effect of *H. perforatum.* Although qRT-PCR and IFA could not detect live viral particles, they were used as a supplement in this study, and in this study we used the method of TCID_50_, which is a general method for the detection of live virus (Leclercq et al., 2014; Petiot et al., 2018). Therefore, the results of antiviral studies *in vitro* are also credible in this study. In addition, although the best way to culture IBV is to use chicken embryos or chickens, the IBV replication in CEK cells was tested in this study. Because if the virus doesn’t replicate well, viral particles can also be reduced or even die, which in turn affects the antiviral effect of the drug. In this study, we showed that IBV can replicate well in CEK cells (**Figure 2**), which is consistent with the research report (Zhang et al., 2018), and HPE had potential utility as antiviral agents against IBV. Our results showed that the HPE can reduce virus mRNA expression level (**Figures 3A** and **8**) and virus titer (**Figure 3A**). Additionally, the SEE had no inhibitory effects and the HPW had poor inhibitory effects (**Figure 3**). The results suggest that the HPE, which contains an enrichment of the main effective substances, was the bioactive fraction of *H. perforatum* extracts.

Moreover, the HPE had anti-IBV activity, such as reducing the fluorescence signal produced by IBV (**Figure 4**), and alleviating the pathological injury of trachea and kidney caused by IBV in chickens (**Figures 6** and **7**). When came to evaluation of antiviral effects, ribavirin was used as control drugs. The inhibitory effect of 78.13 µg/ml HPE was comparable to that of 10 µg/ml ribavirin *in vitro*, and 480 mg/kg HPE was comparable to that of 60 mg/kg ribavirin *in vivo*. In the efficacy of antiviral effects, as in the reporting of others herbs (Choi et al., 2016; Sun et al., 2016; Choi et al., 2017; Yi et al., 2018; Luo et al., 2019), the effects of the herbal extract were lower than ribavirin even under several times higher dosage against IBV mRNA level, virus titer, and histopathological injury caused by IBV. However, it is logical since ribavirin is pure compared to the extracts containing many inactive compounds.

Some studies have shown that *H. perforatum* extract have an antiviral effect on influenza A virus and HIV (Barnes et al., 2001; Birt et al., 2009; Pu et al., 2012), suggesting that HPE have the potential to be developed and used as antiviral drugs. In this study, we found that *H. perforatum* extract had significantly antiviral effect on IBV *in vitro* and *in vivo,* respectively. In addition, anti-IBV effect of HPE might correlate with MDA5 and NF-κB signaling pathway. Belonging to acid inducible gene I (RIG-I)-like receptors (RLRs) in pattern recognition receptors (PRRs), it is well known that MDA5 plays a crucial role in avian respiratory disease progression (Yu et al., 2017), whose function is to identify various pathogen-associated molecular patterns (PAMPs), and activates mitochondrial antiviral signaling gene (MAVS, also called IPS-1/VISA/CARDIF) (Lin et al., 2006), activated MAVS can recruit downstream interferon regulatory factor-3/7 (IRF3/IRF7) (Shi et al., 2015), leading to the rapid production of type I IFNs (Xu et al., 2005; Soulat et al., 2008). NF-κB system is a master transcription factor in the recognition signaling and host responses to immune attacks (Deng et al., 2018). It is well known that NF-κB mediates inflammation, and several cytokine are also linked to NF-κB signaling to enhance the inflammatory responses, such as TNF-α, IL-6, and IL-1β (Salminen et al., 2008).

Several reports have pointed out the MDA5 signaling pathways and innate immune cytokines were activated after infection with IBV M41 strain (He et al., 2016). MDA5 signaling pathway was disrupted by cleavage of the adaptor protein MAVS in the JS/2010/12 strain of IBV infection (Yu et al., 2017). The type I IFN response plays a critical role in resisting SAIBK2 strain of IBV (Yang et al., 2018a). MDA5 signaling pathways and innate immune cytokine (NF-κB and IRF3) were induced after IBV-M41 strain infection (Zhang et al., 2017b). On the other hand, the report had shown that *H. perforatum* extract significantly downregulated the concentration of IL-6 and TNF-α in lung tissue for mice infected with an influenza A virus (Pu et al., 2012). In this study, we found that mRNA expression levels of MDA5, MAVS, IFN-α, IFN-β, NF-κB, TNF-α, and IL-6 were significantly up-regulated after IBV infection *in vitro* and *in vivo* (**Figures 5**, **9**, and **10**). Meanwhile, we found that HPE may be up-regulate mRNA levels of IFN-α and IFN-β by MDA5 signaling pathway, and down-regulate mRNA expression levels of IL-6 and TNF-α through the NF-κB signaling pathway (**Figures 5**, **9**, and **10**), suggesting that HPE may be inhibit IBV by affecting innate immune cytokines.

Because of remarkable antiviral effect on IBV, we analyzed the active components of HPE. The mass spectrum of hyperoside contained a negative quasi-molecular ion at 463.74 m/z [M–H]^−^ (**Figure 12B**), in accordance with the previous literature (Cao et al., 2011). The mass spectrum of **Figure 12B** showed a quasi-molecular ion at 447.44 m/z [M–H]^−^. This corresponds to the fragmentation regularity of quercitrin (Oliveira et al., 2014). Moreover, a quasi-molecular ion [M–H]^−^ at 301.72 m/z confirms the presence of the quercetin (Stierlin et al., 2018) (**Figure 12C**). In addition to a positive quasi- molecular ion at 543.29 m/z [M+Na]^+^ (**Figure 12D**) and a negative quasi-molecular ion [M–H]^−^ at 519.08 m/z (**Figure 12E**), confirming the presence of the pseudohypericin. Finally, the mass spectrum of hypericin shows a positive quasi-molecular ion [M+H]^−^ at 505.44 m/z (**Figure 12F**) and a negative quasi-molecular ion [M–H]^−^ at 503.33 m/z (**Figure 12G**). According to the results in **Figure 3**, the HPE is the main active fraction, containing abundant levels of hyperoside, quercitrin, quercetin, pseudohypericin, and hypericin (**Table 4** and **Figure 11**).

## Conclusions

Our study provides, for the first time, clear evidence that the extract of *H. perforatum*, containing hyperoside, quercitrin, quercetin, pseudohypericin, and hypericin, possess anti-IBV activities. Furthermore, its anti-IBV effect may be associated with reduced mRNA expression levels of the pro-inflammatory cytokines IL-6, TNF-α by NF-κB signaling pathway, and related to up-regulate mRNA expression levels of type I interferon through the MDA5 signaling pathway, and could be useful for the development of new antiviral agents. However, further studies are required to elucidate its detail mechanism of action.

## Data Availability Statement

All datasets generated for this study are included in the article/**Supplementary Material**.

## Author Contribution

HC wrote the manuscript and carried out most of the studies. IM, YZ, and YR participated in the *in vitro* experiments. RZ, XH, LD, HL, XL, XS, and GA participated in the animal experiments. GL designed the experiment and made valuable revision. All authors have read and approved the final version.

## Funding

This research was supported by the National Science and Technology Supporting Projects 31172295 and 31272569 operated by the Ministry of Science and Technology of China. In addition, this project belonged to Key Subject of Traditional Chinese Medicine of Jilin Agricultural Science and Technology University.

## Conflict of Interest

The authors declare that the research was conducted in the absence of any commercial or financial relationships that could be construed as a potential conflict of interest.
