# Supplementary material for: Antiviral Activity Against Infectious Bronchitis Virus and Bioactive Components of Hypericum perforatum L
Source: Front Pharmacol. 2019 Oct 29;10:1272. doi: 10.3389/fphar.2019.01272 (PMC6830131; doi:10.3389/fphar.2019.01272)

**中国科学院植物研究所**

**Institute of Botany, Chinese Academy of Sciences**

**植物标本室**

**Plant Specimen Room**

采集日期： 2012-7-8

Date of collection: July 8, 2012

采集人/采集号： 巴山采集队 6184

Collector/collector number: Bashan Collection team / 6184

采集地: 四川省通江县诺水河保护区狮子口村

Collection place: Shi Zi Kou Village, Nuoshuihe Reserve, Tongjiang County, Sichuan Province

东经： 北纬：

East longitude: North latitude:

生境

Habitat:

海拔 700 m 性状 草本

Altitude: 700 m Character: Herbaceous

高度 m

Height: m

花 黄色

Flower: Yellow

果

Fruit:

中文名 科名 Hypericaceae

Chinese name: Family name: Hypericaceae

学名

Scientific name:

鉴定人（Identification Expert）

鉴定日期（Identification date）

备注（Remark）: 3份（3 Copies）


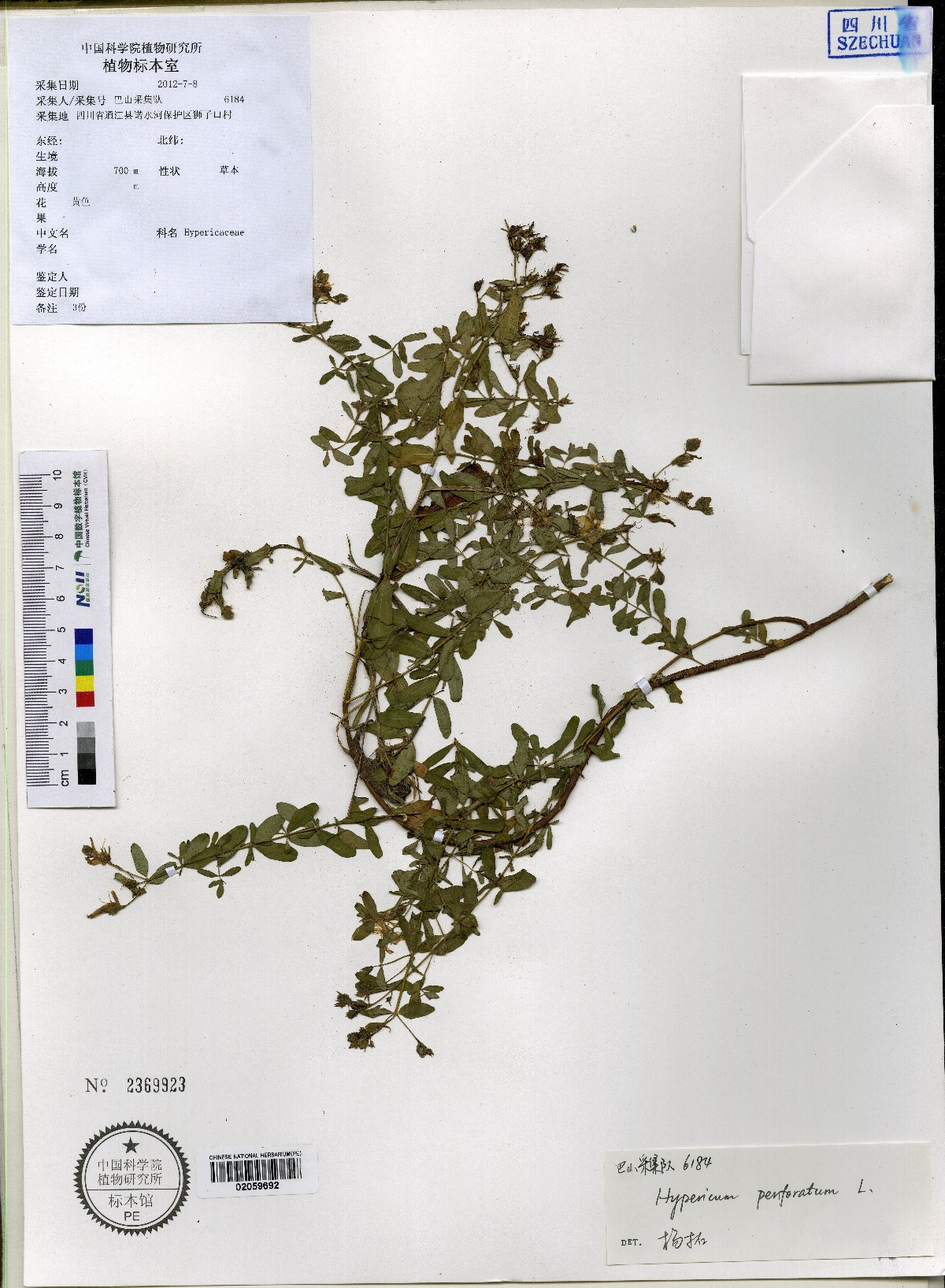

Supplement: Supplementary file 1 [file DataSheet_1.docx]
